# Breaking the silence of female genital schistosomiasis in Ghana’s health system: A case of health workers within the FAST project

**DOI:** 10.1371/journal.pntd.0012443

**Published:** 2024-09-23

**Authors:** Margaret Gyapong, Maxwell Ayindenaba Dalaba, Mustapha Immurana, Alfred Kwesi Manyeh, Kazeem Arogundade, Julie Jacobson, Alison Krentel

**Affiliations:** 1 Institute of Health Research, University of Health and Allied Sciences, Ho, Ghana; 2 Bruyère Research Institute, Ottawa, Ontario, Canada; Universidade do Estado do Rio de Janeiro, BRAZIL

## Abstract

**Background:**

Female Genital Schistosomiasis (FGS) remains a critical and yet neglected topics in Neglected Tropical Diseases (NTDs), significantly affecting the health of women and girls worldwide. Health workers’ knowledge of FGS is vital to the prevention and management of the disease. This study adopted an implementation research approach to identify and address the existing knowledge gap regarding FGS among healthcare workers in Ghana.

**Methods:**

This study was a 3-year (2020–2022) implementation research applying a pragmatic uncontrolled quasi-experimental study design. The study involved a baseline assessment, FGS training intervention for health workers and student nurses, distribution of FGS educational materials, and an endline assessment. A mixed-method approach was applied to data collection involving health workers from two schistosomiasis endemic districts and across the country. NVIVO 12 and STATA 14 were used for qualitative and quantitative data analysis, respectively.

**Results:**

Prior to the intervention, the level of awareness about FGS among health workers was less than 8%, and most participants only understood FGS as merely urogenital schistosomiasis in females. In response to this gap, an FGS education intervention in the form of training of health workers, student nurses alongside the distribution of FGS educational materials were carried out. The intervention enhanced health workers’ awareness of FGS to more than 61%, encompassing an enhanced understanding of the disease’s signs and symptoms to more than 60%, as well as its management strategies. However, access to praziquantel, the primary treatment, remained a significant challenge.

**Conclusions:**

The FGS intervention effectively raised healthcare workers’ awareness and knowledge. Expanding training and improving praziquantel access are essential for optimal FGS management. A multi-faceted approach involving individuals, communities, and the healthcare system is necessary for comprehensive FGS prevention and control.

## Author summary

This study addresses Female Genital Schistosomiasis (FGS), a critical yet neglected issue among Neglected Tropical Diseases (NTDs), particularly impacting women and girls globally. Enhancing healthcare workers’ knowledge of FGS is crucial for effective prevention and management. The research, conducted over three years in Ghana from 2020 to 2022, utilized an implementation research approach.

Initially, baseline assessments revealed alarmingly low awareness (less than 8%) among health workers, who often equated FGS with urogenital schistosomiasis in females. In response, a comprehensive intervention was implemented, including training sessions and distribution of educational materials. This intervention significantly increased awareness among health workers to over 61%, and improved understanding of FGS signs, symptoms, and management strategies to over 60%.

Despite these gains, challenges persist, notably in ensuring access to praziquantel, the primary treatment for schistosomiasis. The study underscores the need for ongoing training initiatives and enhanced medication accessibility to bolster FGS management. It concludes with a call for a holistic approach involving all stakeholders—individuals, communities, and healthcare systems—to advance knowledge and management of FGS effectively.

## Introduction

One of the most prevalent Neglected Tropical Diseases (NTDs) in Sub-Saharan Africa (SSA) is called schistosomiasis, bilharzia or “snail fever.” Schistosomiasis is a parasitic disease that affects men, women and children living in tropical regions. Endemic in 78 countries, this disease affects more than 250 million people with approximately 90% of the disease burden found in Africa [1,2]. Disease transmission occurs through contact with freshwater bodies where aquatic snails breed and releases the parasite that burrows through the skin in contact with water. Regular daily activities (washing, bathing, gathering water, swimming) put adults and children at continuous risk of infection. Current strategies to control schistosomiasis focus on preventive chemotherapy using donated praziquantel [1,2].

When a young girl or woman is infected with schistosomiasis, the worms mate producing millions of eggs every day inside her body. In the case of *S*. *haematobium*, the eggs are released into the genito-urinary tract embedding in the tissue causing inflammation that results in a granulomatous response around the eggs causing Female Genital Schistosomiasis (FGS) [3]. Thus, FGS is a gynaecological disease caused by untreated urogenital schistosomiasis. FGS results in abdominal and pelvic pains, disorders of menstruation, pain during intercourse, vaginal bleeding after intercourse, inflammation of reproductive organs, genital lesions, miscarriage, ectopic pregnancy, infertility and urogenital cancers (bladder cancer) [4].

Increasing evidence shows that a woman with FGS has a threefold increased risk of HIV infection when she becomes sexually active [5–7]. Timely treatment can prevent the disease and reverse some of the associated biological risk factors even in chronic infection [8].

Nonetheless, FGS remains one of the most important neglected areas in women’s and girls’ health globally. An estimated 56 million women have FGS, nearly all in SSA [9]. This infection is a greater concern for women and girls as their gender roles put them at a higher risk of being in contact with infested waters when completing household chores [10].

Schistosomiasis remains a major public health concern in Ghana, especially for residents of poor communities near freshwater sources. These areas often lack access to clean water and proper sanitation facilities. Despite the Ghana Health Service’s efforts in conducting annual mass drug administration programmes and implementing some educational initiatives in schools, schistosomiasis remains prevalent [11]. Moreover, there is a deficiency in educating health workers about schistosomiasis and FGS beyond the standard curriculum (limited training on FGS), leading to a lack of awareness among health workers about FGS and its associated consequences, even in places where the disease is endemic [12,13].

The investigators, therefore, conducted an implementation study to identify and address the FGS knowledge gap among health workers in Ghana. This involved a baseline assessment to examine the knowledge and understanding of FGS among health workers followed by an FGS intervention in the form of training and provision of educational materials to health workers.

## Methods

### Ethical consideration

Ethical clearance for this study was obtained from the Ghana Health Service (GHS) Ethics Review Committee (GHS-ERC 003/04/21) and the University of Health and Allied Sciences Research Ethics Committee (UHAS-REC A.5 [4] 20–21). Written informed consent was obtained from all participants in the study.

### Study design

This was a 3-year (2020–2022) implementation study applying a pragmatic uncontrolled quasi-experimental study design (pre and post-intervention) design. This paper is part of a bigger study called “FGS Accelerated Scale Together (FAST) Transition to scale project: improving women’s health by reducing morbidity from female genital schistosomiasis in Ghana (FAST Project)”. The main aim of the FAST project was to improve adolescent girls and women’s health by reducing morbidity associated with FGS through preventive and curative efforts.

For this aspect of the paper, we report the study activities that involved heath workers in three phases of the study as described below:

### Phase 1: Pre-intervention

This phase was a baseline assessment of the FGS knowledge gap among health workers in the country.

**Fig 1:**
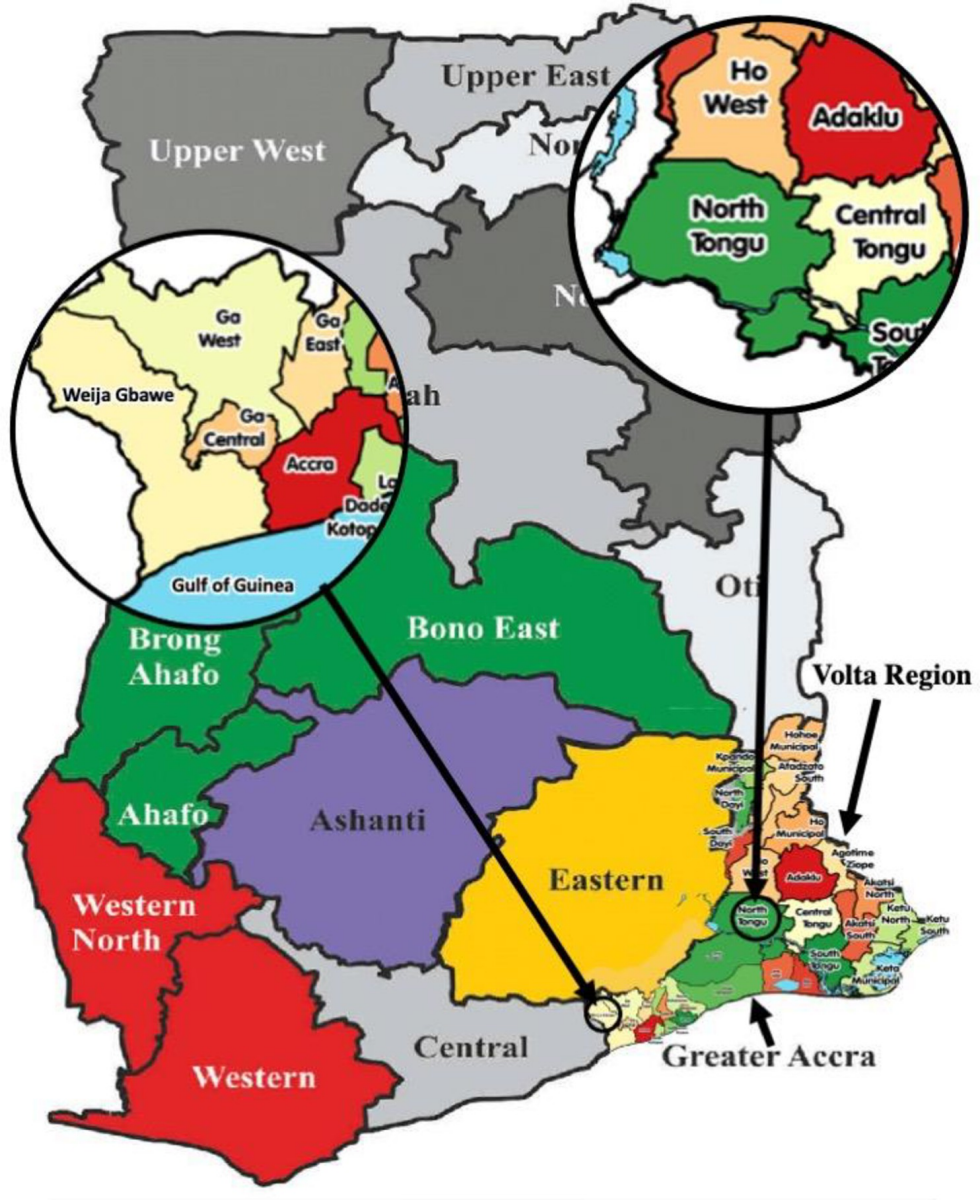
Picture showing Ghana and the study districts (Weija and North Tongu) (http://www.usgs.gov).

Firstly, interviews were held with health workers including Directors of health and Medical Superintendents in two schistosomiasis-endemic districts in Ghana: North Tongu District and the Weija-Gbawe Municipality. Fig 1 shows the map of Ghana and the two selected districts (http://www.usgs.gov).The two districts were selected because they have several communities along the Volta Lake (the largest artificial reservoir in the world) and Wieja Dam (the second largest water reservoir in the country after the Volta reservoir), where the Ghana Health Service reports that schistosomiasis prevalence is at least 80% [14]. Hence, these regions were chosen for the interviews to learn about the knowledge gaps among health workers, which can then be applied to other regions.

Secondly, we engaged relevant stakeholders such as Ghana Neglected Tropical Diseases Programme (NTDP) to identify policy gaps in FGS control. Following the discussions, the Ghana National FGS Committee was established to work towards helping to integrate FGS into routine health services which resulted in the inclusion of FGS in the DHIMS 2 reporting system.

### Phase 2: Intervention

Phase two was the implementation of FGS intervention activities which was based on the baseline assessment. Four main activities were undertaken:

#### Online training of health workers

The FAST Package team, together with the Geneva Learning Foundation, Bridges to Development, conducted an online, interactive, peer-to-peer training on FGS targeting healthcare professionals. The training aimed to equip healthcare providers with essential knowledge about FGS, enabling them to integrate FGS into their clinical practice. This will help to improve women’s reproductive health by decreasing the burden of FGS. Additionally, the training seeks to encourage learners to co-create solutions within their local context to address FGS.

Of the 119 applicants who participated in the online FGS course/training held between May and June 2021, 62 successfully completed the entire session. The participants were a mix of medical doctors, obstetrics and gynecology specialists, nurses/midwives, physician assistants, health information officers, nursing educators, and public health professionals and lecturers from the northern, middle-belt, and southern geographical regions in Ghana.

As part of the training, participants developed feasible action plans to integrate FGS into their practice and establish and maintain awareness in their context. The action plans were designed to reflect actions that could be undertaken without additional budgetary support and within the learners’ own domains of influence.

#### Face-to-face subject matter experts (SME) training

To create a sustainable system of training healthcare providers within the health systems in Ghana, the FAST Package team, carried out a 3 day FGS training in March 2022 to create a cohort of national FGS Subject Matter Experts. Individuals were first identified from the online training programme. These individuals had demonstrated intrinsic motivation to learn about FGS through their online participation. This resulted in a multidisciplinary range of training SMEs including obstetricians / gynecologists, doctors, nurses, midwifes and health information officers. This training had 30 participants.

After the training, participants were given 100 copies (5 copies each) of FGS booklets to distribute to their constituents to help disseminate FGS awareness and management. Expects in the field of FGS, home and abroad, collectively developed the FGS booklet to fit the context of Ghana. The content of the FGS book included the causes, treatment and prevention of schistosomiasis and FGS [9]. All participants (trainees and facilitators) were officially recognized as SMEs on FGS through the awarding of certificates upon completion of the training.

#### Training of frontline health workers on schistosomiasis at endemic districts

Training of frontline health workers (nurses and midwives, based on their involvement in Obstetrics and gynaecology activities) was carried out in two schistosomiasis endemic districts (North Tongu and Weija-Gbawe Municipality). The total number of participants for the training was 116, of which 41 and 75 were from North Tongu and Weija-Gbawe Municipality respectively. After the training, 234 copies of the FGS booklets were distributed to health workers in North Tongu and 429 copies were distributed to health workers in Weija-Gbawe Municipality.

#### Face-to face training of health workers and students

A series of FGS training of health workers and students in the nursing, midwifery and medicine was carried out by the SMEs in their various continuants. For instance, within the period, series of lectures on FGS was delivered to over 300 nursing students, 100 midwifery students and 100 medical students at UHAS, Ho in the Volta Region of Ghana. In addition, lectures on FGS were held for over 600 nursing and midwifery students at Nursing and Midwifery training college (NMTC), Berekum in the Eastern Region of Ghana.

Further, FGS presentations/education were made to about 700 health workers in the eastern region. The participants included: District directors of health, SHEP coordinators, nurses and NTD coordinators.

The Ghana National FGS Committee in collaboration with the Ghana NTDP organised schistosomiasis and FGS face-to-face awareness training for health workers in 15 districts in the Volta, Western and Central regions of Ghana. Over 600 health workers were involved in the training on FGS.

### Phase 3: Post-intervention

Phase three was the evaluation of the intervention. A cross-sectional mixed method approach was applied in data collection involving In-Depth Interviews (IDIs) with health workers as well as quantitative data of health workers in a pre- and post-training assessment format. The interview guide for the IDIs can be found in Supporting information (S1 File).

### Data collection, management and analysis

Graduate-level Research Assistants (RAs) who were trained for five days were used to collect the qualitative data. Interview guides were pretested before the actual data collection. The interviews were face-to-face, and the participants were purposively sampled to include Doctors, Nurses and Midwives based on their involvement in Obstetrics and gynaecology activities. A total of 4 IDIs and 16 IDIs were conducted at baseline and endline respectively.

All qualitative interviews (IDIs) were audio recorded and later transcribed. The transcripts were reviewed for accuracy and completeness. Guided by the objectives of the study and the themes from the transcripts, a codebook was developed. Thematic analysis was carried out using NVIVO 12 software to interpret themes or patterns in the data pertinent to the study aims.

In addition, quantitative data were collected from 116 training participants in the form of pre and post-training evaluations to determine the changes in FGS knowledge among the health workers. The participants (health workers) were nurses/midwives identified to be involved in obstetrics and gynaecology activities in their various health facilities in the schistosomiasis endemic districts of North Tongu and Weija-Gbawe districts. The quantitative data were collected using a paper-based questionnaire and then entered STATA 14.0 for cleaning and analysis. The data were analysed using simple frequencies and percentages.

## Results

This section presents quantitative results from pre- and post-training evaluations of health workers (nurses/midwives) from North Tongu and Weija-Gbawe districts (116 respondents) as well as online training evaluation across health workers in Ghana (62 respondents).

The section also presents the qualitative findings from baseline and endline IDIs from North Tongu and Weija-Gbawe districts (20 participants).

### Socio-demographic characteristics

Table 1 presents the sociodemographic characteristics of frontline Health workers from the North Tongu and Weija-Gbawe districts who participated in the qualitative interviews (IDIs). The majority of the participants at the endline were females and the average age of the participants at baseline and endline was 34 and 48 years respectively.

**Table 1:** Sociodemographic characteristics of IDI participants.

	Endline		Baseline	
Variable	Freq (16)	Percent (100%)	Freq (4)	Percent (100%)
***Sex***				
Female	14	87.5	2	50
Male	2	12.5	2	50
***Rank***				
Community Health Nurse (CHN)	3	18.8	0	0
Medical Officer	3	18.8	2	50
Medical Superintendent	1	6.3	2	50
Midwife	2	12.5	0	0
Nurse	5	31.3	0	0
Physician Assistant	2	12.5	0	0
***Education***				
Degree	6	37.5	0	0
Diploma	7	43.8	0	0
Masters	3	18.8	4	100
**Average *age***	34		48	
**Average *number* of years of services**	5		8	

### Awareness about schistosomiasis and FGS

In the quantitative assessment in the two schistosomiasis endemic districts, the results showed that before FGS training, health workers’ level of awareness (very aware) about FGS was less than 8% (North Tongu 7.3%; Weija-Gbawe 4.1%).

**Fig 2:**
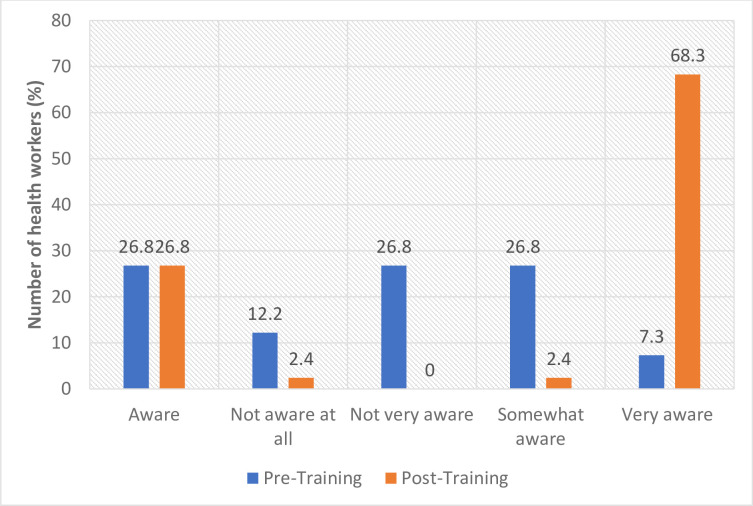
Awareness about schistosomiasis and FGS (North Tongu).

**Fig 3:**
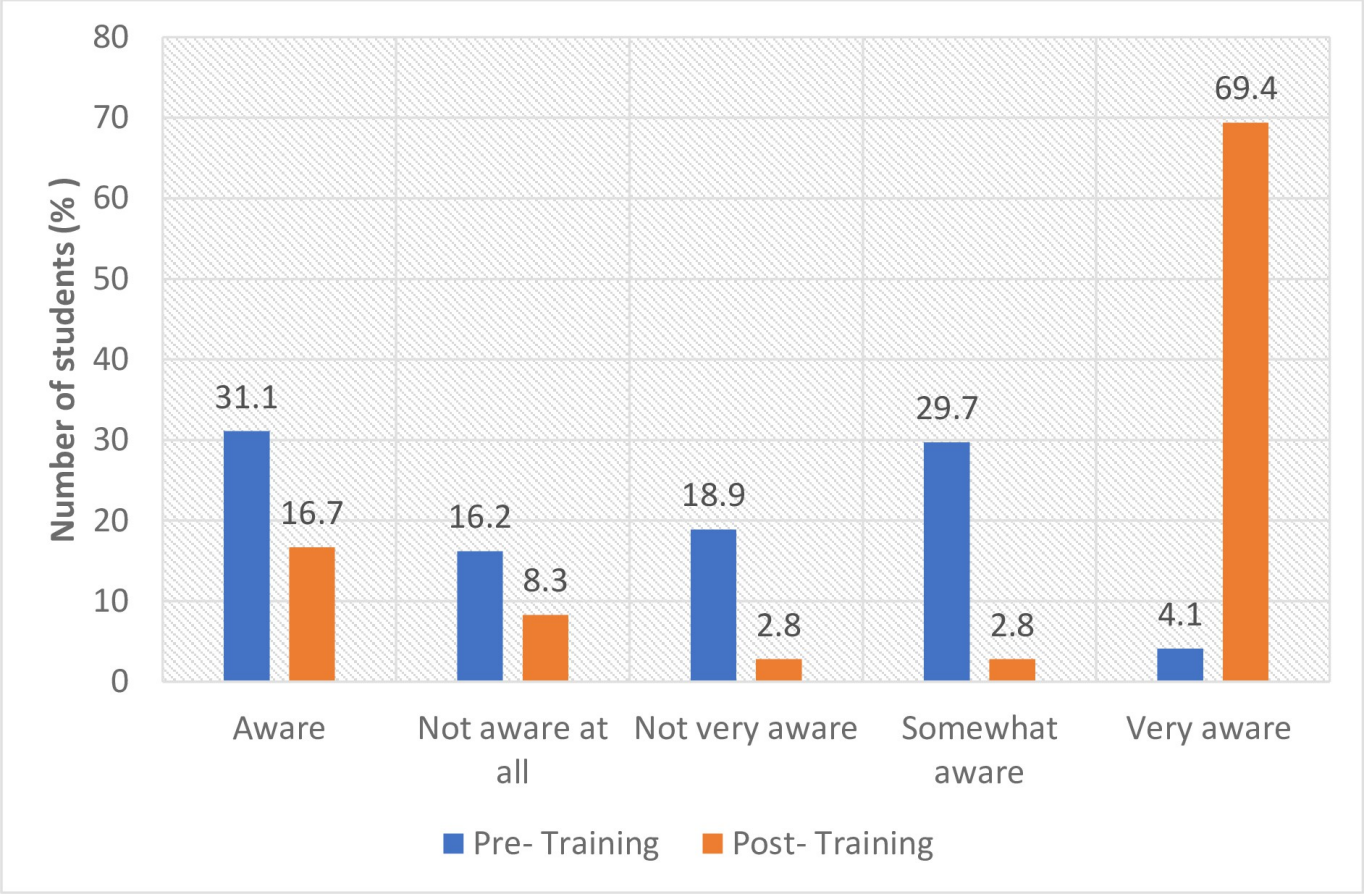
Awareness about schistosomiasis and FGS (Weija).

Following the FGS training, awareness significantly improved and more than 68% (North Tongu 68.3%; Weija-Gbawe 69.4%) of the participants mentioned that they were now very aware of FGS (Figs 2 and 3), representing more than 61% improvement in the level of level of awareness of FGS.

Similarly, in the qualitative assessment, the majority of the health workers who participated in the baseline and endline assessments indicated that they had heard of schistosomiasis. However, with regard to FGS, the knowledge level was low before the FGS training intervention. Furthermore, some of the health workers confessed that before the FGS training, they were not aware that women can get schistosomiasis and therefore they did not know about FGS prior to the training. They therefore acknowledged that the FGS intervention (FGS training and FGS booklet) has improved their competencies around FGS. These results suggest that the FGS training was effective in raising health workers’ awareness of FGS.

*“Before this training, I knew nothing about FGS. What I knew about was the normal schistosomiasis (IDI-Health worker, North Tongu, Endline)”*

*“At first*, *I didn’t know females get schistosomiasis*. *I knew about only the males*. *However*, *after the training*, *I am now aware of FGS…However*, *I did not get any FGS case after the training yet*. *In fact*, *I told one of my colleagues that I missed an FGS case because I encountered a lady before the FGS training and all the complaints she presented to me were related to FGS but because I was at that time not aware of FGS*, *I think a missed her*. *And there is no way I could trace her up (IDI-Health Worker*, *Weija-Gbawe*, *Endline)*.

### Health workers knowledge in FGS diagnosis

Following the implementation of a FGS training intervention, there was a significant increase in healthcare workers’ ability to diagnose FGS. In North Tongu, the percentage of health workers reporting confidence in FGS diagnosis rose from 26.8% to 97.6%, demonstrating an impressive 70.8% improvement. Similarly, in Weija-Gbawe, the proportion of confident healthcare workers increased from 35.4% to 95.8%, representing a notable 60.4% rise (Table 2). These findings illustrate the positive impact of training on healthcare workers’ capacity to diagnose FGS effectively. This improved diagnostic ability will ultimately lead to earlier detection and treatment of FGS, contributing to improved patient outcomes and enhanced public health efforts.

**Table 2:** Knowledge to diagnose, record and treat FGS.

	North Tongu		Weija-Gbawe	
	Pre-Training	Post-Training	Pre- Training	Post- Training
**Knowledge to diagnose FGS**				
No	30 (73.2)	1 (2.4)	42 (64.6)	3 (4.2)
Yes	11 (26.8)	40 (97.6)	23 (35.4)	69 (95.8)
Total	41 (100)	41 (100)	65 (100)	72 (100)
**Knowledge to treat FGS**				
No	30 (73.2)	2 (4.9)	48 (75)	0 (0)
Yes	11 (26.8)	39 (95.1)	16 (25)	71 (100)
Total	41 (100)	41 (100)	64 (100)	71 (100)
**Knowledge to refer FGS**				
No	24 (58.5)	0 (0)	26 (39.4)	4 (5.7)
Yes	17 (41.5)	41 (100)	40 (60.6)	66 (94.3)
Total	41 (100)	41 (100)	66 (100)	66 (100)
**Knowledge to record FGS**				
No	28 (68.3)	0 (0)	29 (44.6)	0 (0)
Yes	13 (31.7)	41 (100)	36 (55.4)	71 (100)
Total	41 (100)	41 (100)	65 (100)	71 (100)
**Knowledge to prevent FGS**				
No	22 (53.7)	0 (0)	24 (38.1)	0 (0)
Yes	19 (46.3)	41 (100)	39 (61.9)	71 (100)
Total	41 (100)	41 (100)	63 (100)	71 (100)

### Health workers knowledge to diagnose, treat and record FGS in DHIMS 2 System

The District Health Information Management System–2 (DHIMS 2) serves as the central database for capturing disease data within the healthcare system. Following the study intervention, FGS was integrated into the DHIMS 2 reporting system in 2021 alongside urogenital schistosomiasis.

To facilitate accurate FGS case identification and reporting, health workers underwent training. Participants reported that the training effectively equipped them with the necessary knowledge and skills. Consequently, the ability to identify and record FGS cases improved significantly (100%, Table 2).

DHIMS 2 data analysis revealed that FGS case reporting is ongoing. In 2022 and 2023, a total of 147 and 69 FGS cases, respectively, were recorded (Table 3).

**Table 3:** Records of Schistosomiasis and FGS in the DHIMS 2 data.

Region	Schistosomiasis (2022)	Schistosomiasis (2023)	Female Genital Schistosomiasis (2022)	Female Genital Schistosomiasis (2023)
Ahafo	20	4	0	0
Ashanti	548	294	14	11
Bono	15	110	0	0
Bono East	259	384	13	17
Central	249	222	6	3
Eastern	543	473		12
Greater Accra	375	525	91	7
North East	5	2	0	0
Northern	26	37	0	4
Oti	99	70	0	0
Savannah	44	85	0	0
Upper East	82	135	0	3
Upper West	79	46	1	1
Volta	175	267	13	9
Western	191	150	9	1
Western North	60	50	0	1
**Ghana**	**2770**	**2854**	**147**	**69**

### Knowledge of FGS from online training

**Fig 4:**
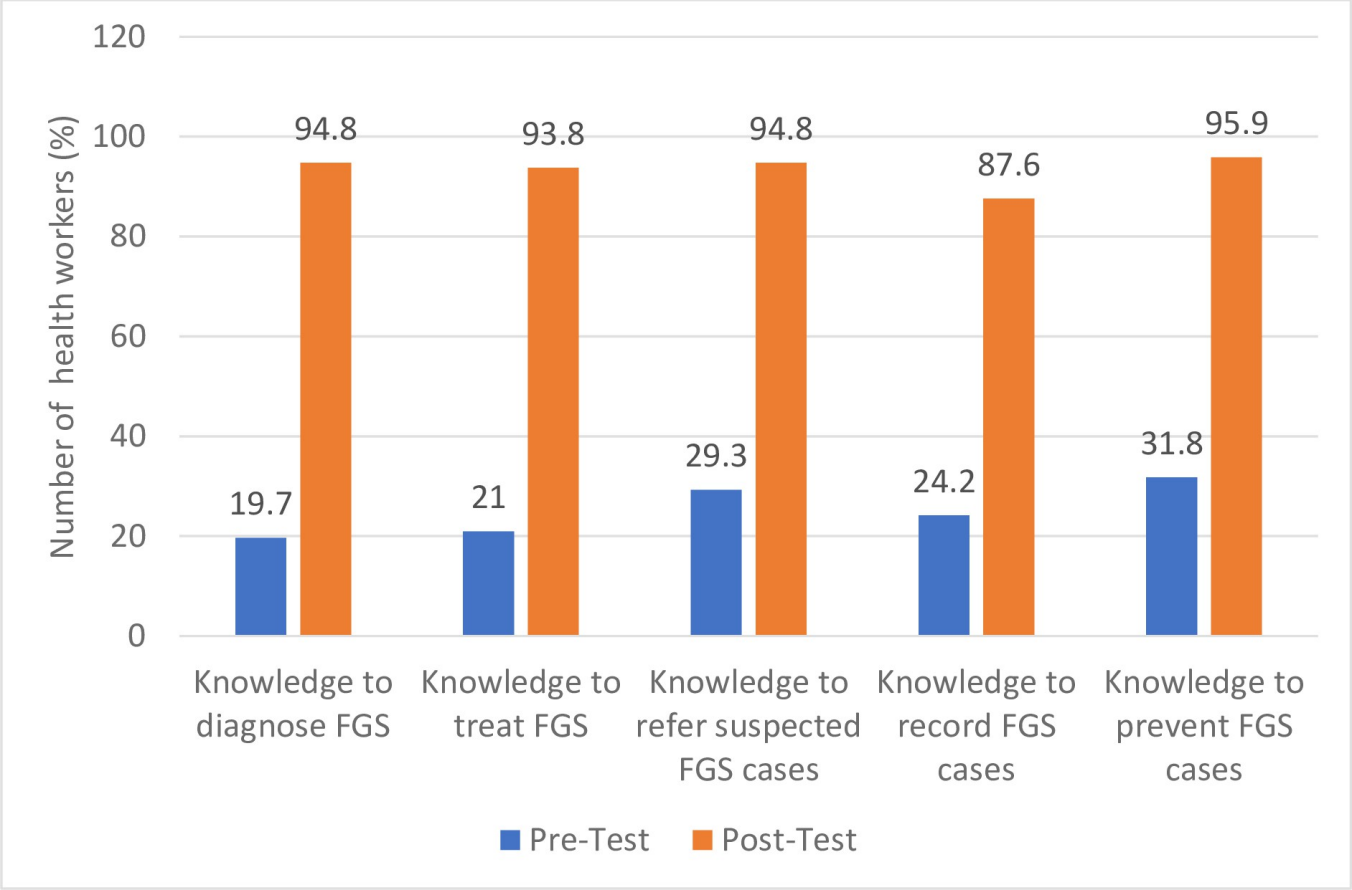
Knowledge of FGS from Online Training.

Similarly, pre- and post-training evaluation of the FGS online training showed improvement in knowledge to diagnose, treat, record and prevent FGS (Fig 4). For instance, there was a 75.1% increase in knowledge to diagnose, amongst those who completed the course (Fig 4).

### Scarcity of praziquantel hamper schistosomiasis and FGS treatment efforts

Healthcare workers reported encountering difficulty in obtaining praziquantel, often finding it unavailable at both health facilities and pharmacies. This scarcity forced them to adopt alternative approaches, such as occasionally resorting to antibiotics as a treatment option. In some cases, they would prescribe praziquantel to patients, leaving them to independently search for and purchase the medication at pharmacies, where its availability was uncertain.

*“When they come to the health facility and we do not have praziquantel at the facility, I prescribe it to them to go buy from the pharmacy shop. Mostly we do not have praziquantel in the health facilities (IDI, Health Worker, Weija-Gbawe, Endline)”*

*“I think some of my colleagues sometimes give them antibiotics and painkillers if there is no praziquantel*. *Sometimes*, *after MDA*, *they give us the remaining praziquantel*. *If a patient comes and fortunately we have some*, *then we can give the praziquantel (IDI*, *Health Worker*, *North Tongu*, *Endline)”*

### FGS health seeking

Health care workers highlighted the occurrence of delayed care-seeking for schistosomiasis among women, often due to a lack of awareness about the disease and its potential progression to FGS. These women often remain unaware of their infection until they present for antenatal care (ANC) services. Notably, during ANC examinations, trained health workers can identify suspected cases of schistosomiasis/FGS. This was exemplified by a medical superintendent who recounted an instance where he implemented routine testing for schistosomiasis during ANC, leading to the identification of multiple cases.

*“the very young ones do not know how they contracted it (schistosomiasis)…like the routine tests that we do for the pregnant women, occasionally some of them test positive for schistosomiasis. I think we discharged a woman treated for FGS a month ago. I think we were just doing routine testing and then, schistosomiasis ova was present…”(IDI, medical superintendent, North Tongu).*

The cost of treatment for schistosomiasis and FGS presents a barrier to seeking appropriate care, particularly for vulnerable populations. Healthcare workers reported that many community members either forgo treatment entirely or resort to low-cost alternatives due to financial constraints. These alternatives, such as herbal remedies or delaying treatment until mass drug administration (MDA) campaigns, may not be effective and can lead to further complications. The high cost of treatment can be attributed to the scarcity of praziquantel, the primary medication for schistosomiasis and FGS, at both health facilities. This scarcity often forces individuals to seek treatment at chemist shops, where prices are typically higher.

*“Sometimes due to cost, parents do not treat the child when the child has schistosomiasis. They wait till it is time for MDA because, during that period, they can get it for free (IDI-Health worker, North Tongu, Baseline)”*

## Discussion

Gender roles expose females to schistosomiasis and FGS and therefore the need for strategic interventions, particularly in schistosomiasis endemic communities. The study sought to use an implementation design to identify and address the FGS knowledge gap among health workers in Ghana.

The findings of the study revealed that the level of awareness of urogenital schistosomiasis as well as some common symptoms of schistosomiasis such as blood in the urine was high among health workers. However, it was observed that at the time of baseline assessment and before FGS training intervention, very little was known about FGS awareness, diagnosis and treatment. This finding is in tandem with previous studies in Tanzania and Nigeria that reported high knowledge about schistosomiasis among healthcare workers but limited knowledge about FGS [15,16]. FGS is frequently given little attention in medical education and continues to be poorly understood by health workers, which commonly results in FGS being misdiagnosed or mistakenly diagnosed as an STI or cervical cancer and then given improper treatment [9]. Traditionally, urogenital schistosomiasis has been primarily associated with male symptoms and this focus on male-specific manifestations has led to the neglect of FGS, which primarily affects women and often presents with less obvious symptoms [17,18].

Nonetheless, due to the study intervention, the level of awareness and knowledge on diagnosis and treatment of FGS among health workers was reported to have improved within the period, demonstrating the impact of the intervention. Furthermore, health workers who did not participate in the FGS intervention will gain awareness about FGS. This is because the training adopted a "trainer of trainers" approach, where participants received FGS materials to disseminate among their colleagues, thus enhancing overall understanding of FGS. Continuous FGS education among health workers is very important in controlling FGS. The Ghana National FGS Committee mentioned that they would continue with the in-service training of nurses across the country and will also engage the Nurses and Midwifery Council concerning curriculum revision to incorporate FGS diagnosis and management. With these initiatives, FGS discussion will be reinforced at the school and practice levels. Improved competency about FGS among health workers will facilitate the prevention, diagnosis and treatment/management of FGS and therefore calls for more efforts to sensitize health workers about FGS beyond this study.

Understanding diseases is crucial for accurately capturing data into DHIMS 2 to assess their prevalence and to make policy decisions [19]. The study’s findings indicated that the intervention enhanced both knowledge and skills, enabling better identification and precise documentation of FGS cases in the DHIMS 2 system. These acquired data-capturing abilities will lead to a more precise understanding of FGS prevalence, facilitating targeted interventions and enhanced patient care.

Although praziquantel is the recommended medication for the treatment and prevention of schistosomiasis and FGS in Ghana [20–23], access and use of the medication is a challenge. Currently, praziquantel is only available during MDA and sparsely available in health facilities and pharmacies/drugs shops. However, this contradicts the latest WHO guidelines, which advocate for praziquantel’s availability at health facilities for treating all infected individuals, regardless of age, including pregnant women beyond the first trimester, lactating mothers, and children under two years old [24]. This underscores the necessity for the Ghana Health Service to intensify collaboration and actively pursue the integration of schistosomiasis management into the healthcare system.

Furthermore, the cost of praziquantel poses another challenge, as it is often unaffordable for community members purchasing from pharmacies/drug shops. For instance, the price of a single 600mg tablet of Praziquantel is about GHc50 (USD5) and given that praziquantel is prescribed at a single dose of 40 mg per kilogram of body weight [9,20], if a child weighs 45kg, he or she will require as many as three tablets. The cost of treatment with praziquantel can be overwhelming if a complete dose is considered, as single dose is more than three times the daily minimum wage (GH 14.88) [25] of a worker in Ghana. These findings highlight the need for continued efforts to address supply chain issues to ensure effective and accessible treatment for FGS patients [26].

### Limitations of the study

#### Selection bias

Training frontline health workers on schistosomiasis in endemic districts was restricted to nurses and midwives due to their involvement in obstetrics and gynecology. While this choice aligns with the focus on FGS diagnosis, it excludes other healthcare providers, such as general practitioners and pediatricians, who may also encounter FGS patients. However, we assumed that these excluded professionals possess sufficient knowledge about FGS due to their higher professional status and therefore will not affect the study findings.

#### Limited sample size

The relatively small number of trained health workers may restrict the study’s impact to a limited population, potentially affecting its representativeness at the national level. To mitigate this, trained personnel were encouraged to disseminate their knowledge to colleagues.

#### Outcome measurement

While the study effectively assessed changes in awareness and knowledge, it did not directly measure the impact on FGS diagnosis and treatment practices. This limits our ability to fully evaluate the intervention’s effectiveness in improving patient care. Nevertheless, it is anticipated that participants will apply their newfound knowledge in practice.

## Conclusion

There has been a lack of awareness and knowledge about Female Genital Schistosomiasis (FGS) among healthcare workers, leading to missed diagnoses and inadequate treatment. While the study intervention has improved some health workers awareness and the knowledge to diagnose and treat FGS, continued efforts are crucial to achieving greater impact.

We therefore recommend the following to expand FGS knowledge and awareness:

- Curriculum Integration: Incorporating FGS diagnosis and management into the training curriculum for healthcare workers at all levels is essential for equipping them with the necessary skills to identify and treat the disease.
- Equipped Facilities: Ensuring health facilities are adequately equipped with praziquantel, medical equipment, and supplies is crucial for effective diagnosis and treatment of FGS.
- Continued training and refresher courses: Providing ongoing training and refresher courses for healthcare workers helps ensure they stay up-to-date on the latest FGS information and treatment protocols.
- Public awareness campaigns: Raising public awareness about FGS symptoms, risk factors, and available treatment options is crucial for encouraging people to seek timely medical attention.
- Community Engagement: Engaging community leaders and health workers in raising awareness and promoting FGS prevention strategies can contribute significantly to reducing the disease burden.
- Collaboration and Partnerships: Fostering collaboration and partnerships between healthcare systems, research institutions, and NGOs can accelerate progress toward FGS elimination.
- Comprehensive approach to FGS: Holistic approach to FGS: FGS elimination should be achieved through a comprehensive and holistic programme package covering all community members (males, females and children) at risk of contracting the disease.

## Supporting information

S1 File Indepth Interview(IDI) guide. (DOCX)
