# Supplementary material for: Breaking the silence of female genital schistosomiasis in Ghana’s health system: A case of health workers within the FAST project
Source: PLoS Negl Trop Dis. 2024 Sep 23;18(9):e0012443. doi: 10.1371/journal.pntd.0012443 (PMC11449374; doi:10.1371/journal.pntd.0012443)
Supplement: S1 File — (DOCX) [file pntd.0012443.s001.docx]

**In-Depth Interview Guide for endline data collection (Health Care Providers)**

*Key informants:* health care providers, e.g. clinicians, nurses and midwives

Begin interview after introducing yourself, reading the information sheet and the informed consent sheet has been read and signed.

- - 1. **GENERAL INTRO QUESTIONS**

**Thank you once again for taking the time to speak with me today.**

**As we get started, I’d like to know a little bit more about this district / community. What can you tell me about it?**

***Probe with:***

- Primary occupations of people living here
- Any special cultural festivals
- Languages

**What are the main health concerns people have here?**

***Probe with:***

- Seasonality? (rainy season / dry season)
- Part of the community or the whole community affected?
- History of this health concern (new concern? Been here for a long time?)
  - 1. **QUESTIONS ABOUT ROLE WITHIN THE HEALTH SYSTEM**

**Can you tell me about your current role within the health system?**

***Probe with:***

- Length of time in this role?
- Areas of particular interest?
- Educational background?
  - 1. **QUESTIONS ABOUT SCHISTOSOMIASIS**

**I understand that there is schistosomiasis (use local name) in this district. Can you tell me a bit more about how people in this district perceive this disease?**

***Probe with:***

- Local names (different for girls / women and boys / men)
- How do people think you get it? (If respondent mentions water, prompt with: what in the water causes disease?)
- Who people think gets it most? (prompt with girls, boys, women, men) do you agree?
- Do you know anyone who has schistosomiasis?
- Is it a problem here?

**When someone gets infected with schistosomiasis, what happens to them in this district?**

***Probe with:***

- How do they know they are infected?
- Symptoms different for girls and boys? Why?
- Length of time that the symptoms last?

**One of the symptoms of schistosomiasis infection is blood in the urine. Can you tell me what people in this district say about this symptom and where it comes from?**

***Probe with:***

- Is it different for boys and girls?
- Cultural explanations
- How long does it last?
- Can it be treated?
- Do the students say something different?

**Please describe for me what people do if they experience blood in their urine.**

***Probe with:***

- Where do they go - Health provider – who? Traditional healer?
- Any home remedies?
- Do nothing at all
- Is this different for boys and girls?
- Do the students do something different?

**I’d like to talk about schistosomiasis infection in girls and women specifically. Can you talk about any ways that girls or women may be affected differently than boys with schistosomiasis?**

***Probe with:***

- Symptoms?
- Treatment options?
- Stigma related to symptoms

**I’d like to talk about Female Genital Schistosomiasis. Can you tell me what you understand about FGS?**

***Probe with:***

- Is this something you have heard about?
- Symptoms?
- Stigma related to symptoms
- Common disease for girls in your school / women in your community?

**If a woman or girl has FGS, what treatment options are available to them in this district?**

***Probe with:***

- Health provider? Midwife? Nurse?
- Diagnosis available?
- Cost?
- Availability of praziquantel at the local health care facility outside of MDA?
- Any stigma associated with treatment seeking?
- Has this improved / changed in the last year?

**Can you describe the level of knowledge about schistosomiasis and FGS amongst health care providers in your district?**

***Probe with:***

- Health provider? Midwife? Nurse? Doctor? Specialists?
- Diagnostic training
- Training on treatment options
- Stigma?
- Any new training?

**(ENDLINE QUESTIONS)**

**I’d like to talk about training specific to FGS that was offered in this district for health care providers. Please tell me what you have heard about FGS-specific training.**

***Probe with:***

- What was/were the training you participated in?
- Prompt with FGS Online training (July 2021), Face to Face Training (October 2021- February 2022), Subject Matter Expert Training (February – March 2022), Impact Accelerator Event (online – November 2021)
- Content?
- Online format? District?
- Duration of training?
- Participation – yes / no – why not?

Have you participated in any of the trainings that have been offered?

Probe with:

If they did, which ones? what motivated you to participate?

If they did not participate, GO TO BOX BELOW?

**If respondent did participate, please tell me what you think about the training you received.**

***Probe with:***

- Relevance to their training capacity / level
- Ability to participate in the online format / Ability to participate in the district training?
- Format of the training – online, peer groups, materials, contact with other learners
- How the training (district and online) could be improved?

**Can you tell me about any way your diagnosis and treatment practices changed after this training?**

***Probe with:***

- Have you seen or diagnosed any cases?
- Diagnosing behaviour for FGS changed?
- Availability of Praziquantel?
- Difference between girls and women?

**Is there a specific patient with FGS you remember that you’d like to tell me about? What happened?**

***Probe with:***

- Presenting symptoms
- Diagnosis – how was this done
- Treatment – paid or unpaid?
- Stigma related to the symptoms?
- Resolution of symptoms after treatment?
- Any members of the family treated as well? (contact tracing)

Any positive or negative changes that you have experienced from your involvement in the training?

**Of these changes can you describe the most significant change that you have experienced that resulted from your involvement with the training?**

***Probe with:***

- Why is this story significant to you?
- What do you think brought about the change?

**If respondent did NOT participate in the FGS training, please tell me why you did not participate?**

- Would you want to participate in a training?
- What would make it possible or interesting for you to take part in a training?

**Would you like to participate in it if it were offered again? Why? Why not?**

- - 1. **QUESTIONS ABOUT SCHOOL-BASED OR COMMUNITY-BASED MDA**

**In this next section of questions, I’d like to talk a bit about the distribution of tablets for schistosomiasis in your district.**

**Can you tell me about the school-based or community-based distribution programme?**

***Probe with:***

- What did you understand about the distribution?
- When did it happen?
- What did you do? Your role?
- What were people talking about in the community when the distribution took place?
- What were the students talking about in school when the distribution took place?

**What about the children who are not in school, how do they access treatment?**

**I’d like us to talk a bit more about the good and bad parts about the schistosomiasis pills (or Praziquantel).**

**Let’s think about the good things first.**

***Probe with:***

- What do you think is good about the treatment?
- How does it help the community?

**Let’s do the same for the bad things.**

***Probe with:***

- What do you think is bad about the treatment?
- How does the availability of food impact acceptability of treatment?
- Do you know anyone who suffered in some way after taking the treatment? What happened?

**Let’s talk about the pills themselves a bit. Can you tell me about how the community reacts to the pills?**

***Probe with:***

- Difficulty swallowing the tablets? Ways to mitigate choking?
- Anyone who refuses to participate?

**Please describe any information community / schools were given about the distribution.**

***Probe with:***

- Information leaflets
- Posters in the schools
- Information for the parents
- Teaching curriculum
- FGS Educator’s booklets

**What recommendations do you have to make the school-based or community-based distribution better next year?**

***Probe with:***

- Training for teachers
- Information for students / parents
- Water / snack to help students take the treatment
- More information about side effects
  - 1. **QUESTIONS ABOUT COMMUNITY ENGAGEMENT**

**In this last set of questions, I’d like to talk about community engagement and understand your recommendations to improve the diagnosis and treatment for women and girls with FGS.**

**How do women and girls in your community know that there is care (diagnosis and treatment) available for FGS?**

***Probe with:***

- Differences for women and girls?
- Has there been a change in the number of women and girls coming to clinic? (If relevant) Why do you think there has been a change?
- Who is a trusted source of information for FGS? Different for women and girls?
- Any stigma?
- Barriers to treating schistosomiasis or FGS in women and girls?

**In your opinion, has there been any change in the uptake of school-based or community-based MDA with Praziquantel over the last 12 months. Why / why no change?**

**Please tell me how community supports the school-based MDA. Please describe the current level of support and cooperation in the community for the school-based distribution?**

***Probe with:***

- What are opportunities for community involvement?
- What are the barriers?
- Do we need different approaches for boys and girls?

**What recommendations would you make to improve the engagement of community members?**

***Probe with:***

- Any specific groups that may be hard to reach?
- Specific materials?
- Specific messages about water access?
- Improving overall levels of sanitation (e.g. people not urinating in the rivers / ponds)
- Availability of diagnosis and treatment

**Is there anything else you would like to add?**

***Thank you for your time!***

**Please probe continually throughout with things like:**

- Why did you say that?
- What do you mean by that?
- Could you tell me a bit more?
- Can you say what you mean by that?
- So then what happened?
- Oh, that makes sense…
- That’s interesting…
