# Supplementary material for: Breaking the silence of female genital schistosomiasis in Ghana’s health system: A case of health workers within the FAST project
Source: PLoS Negl Trop Dis. 2024 Sep 23;18(9):e0012443. doi: 10.1371/journal.pntd.0012443 (PMC11449374; doi:10.1371/journal.pntd.0012443)
Supplement: Attachment — Submitted filename: PNTD-D-24-00259_reviewer Ghana Project Reviewer One.pdf [file pntd.0012443.s002.pdf]

Reviewer One Comments

Line 60: Introduction

Please include some statements on the activities of the current schistosomiasis control programme in the area and indicate the shortcomings that have led to a lack of awareness about FGS and its poor management. Many endemic countries have school schistosomiasis control programmes. Is it the same in Ghana? If so, why should awareness be so low in a disease-endemic region? If the school programmes exist, are the girls included in the interventions? What is the involvement of the Ghana FGS Committee and the GNTP?

The absence of praziquantel in public health facilities in schistosomiasis regions is a critical observation that speaks to the disease programme's inadequacy. This needs to be elaborated on.

Line 76: More complications of schistosomiasis could be included, especially urogenital cancers such as bladder.

Line 192; Ethical considerations

A study like this one that is likely to influence policy change would normally receive additional approvals from the Ministry of Health. If the authors could mention that, it adds credence to the paper. Also, include a statement that participants volunteered to respond to the study's requirements.

Line 199-202: It would enrich the text (especially for international readers) to **include a map** indicating the location of the study districts where schistosomiasis is endemic in Ghana.

Line 209: Table one

1. Consistency in decimal placement would improve the quality of the table. One decimal place for percentages is enough to convey the message.
2. CHN should be in full to enhance the understanding of the table contents and relate to the text.

Line 226 Figure One

1. It is concerning that some health workers remained unaware of FGS even after training. Therefore, explanations should be given for this peculiar finding. Is it possible that health workers who did not undergo training, also became more aware about FGS through their peers?

Line 347: The sentence is unclear. This could be due to a typo or an omission that caused an unintended grammatical error. Please correct that sentence.

Lines 361-362: Treatment of schistosomiasis in females and males, including children. Young children who normally accompany females during their daily chores in infested water bodies are also at risk of infection, and mentioning that fact in the recommended actions would add more information and enrich the paper.

Lines 376-378: Inclusion of statements on advocacy for a comprehensive schistosomiasis control programme that is holistic and includes boys and girls (FGS), schools and the community

Lines 385-387: As stated above, FGS elimination should be achieved through a comprehensive and holistic programme package covering all community members (males and females) at risk of contracting the disease.
