# Supplementary material for: Breaking the silence of female genital schistosomiasis in Ghana’s health system: A case of health workers within the FAST project
Source: PLoS Negl Trop Dis. 2024 Sep 23;18(9):e0012443. doi: 10.1371/journal.pntd.0012443 (PMC11449374; doi:10.1371/journal.pntd.0012443)
Supplement: Attachment — Submitted filename: PNTD-D-24-00259 FGS manuscript in Ghana.pdf [file pntd.0012443.s003.pdf]

PLOS Neglected Tropical Diseases

Breaking the silence of female genital schistosomiasis in Ghana's health system: A case of health workers within the FAST project

--Manuscript Draft--

**Manuscript Number:**	PNTD-D-24-00259
**Full Title:**	Breaking the silence of female genital schistosomiasis in Ghana's health system: A case of health workers within the FAST project
**Short Title:**	Health workers knowledge about Female genital schistosomiasis in Ghana.
**Article Type:**	Research Article
**Keywords:**	Neglected Tropical Diseases, Schistosomiasis, Female Genital Schistosomiasis, Mass Drug Administration, Ghana
**Abstract:**	Background: Female Genital Schistosomiasis (FGS) remains one a critical and neglected topics in Neglected Tropical Diseases (NTDs) and the health of women and girls worldwide. Health workers' knowledge of FGS is vital to the prevention and management of the disease. This study conducted implementation research to identify and address the FGS knowledge gap among health workers in Ghana.<br>Methods: This study was a 3-year (2020 -2022) implementation research applying a pragmatic uncontrolled quasi-experimental study design. The study involved a baseline assessment, an intervention phase involving the training of health workers about FGS and an endline assessment. A mixed-method approach was applied to data collection involving health workers in two schistosomiasis endemic districts and health workers across the country. NVIVO 12 and STATA 14 were used for qualitative and quantitative data analysis, respectively.<br>Results: Prior to the intervention, the level of awareness about FGS among health workers was less than 8%, and most participants only understood FGS as merely urogenital schistosomiasis in females.<br>In response to this gap, an FGS education intervention in the form of training of health workers, student nurses alongside the distribution of FGS educational materials were carried out. The intervention enhanced health workers' awareness of FGS to more than 61%, encompassing an enhanced understanding of the disease's signs and symptoms to more than 60% , as well as its management strategies.<br>Nevertheless, a key challenge remains in ensuring access to praziquantel, the primary medication for treating and preventing schistosomiasis.<br>Conclusions: The FGS intervention has improved health workers' awareness and understanding of FGS. However, there is still a need for expanded training of health workers as well as improvement in the access to praziquantel to facilitate FGS management. In addition, a holistic strategy encompassing all stakeholders at the individual, community, and health-system levels is required to improve the general knowledge and management of FGS
**Additional Information:**	
**Question**	**Response**
**Government Employee**<br><br>Are you or any of the contributing authors an employee of the United States government?<br><br>Manuscripts authored by one or more US Government employees are not copyrighted, but are licensed under a CC0 Public Domain Dedication, which allows unlimited distribution and reuse of the	No - No authors are employees of the U.S. government.

Powered by Editorial Manager® and ProduXion Manager® from Aries Systems Corporation

article for any lawful purpose. This is a legal requirement for US Government employees.<br><br>This will be typeset if the manuscript is accepted for publication.	
**Financial Disclosure**<br><br>Enter a financial disclosure statement that describes the sources of funding for the work included in this submission. Review the submission guidelines for detailed requirements. View published research articles from *PLOS Neglected Tropical Diseases* for specific examples.<br><br>This statement is required for submission and **will appear in the published article** if the submission is accepted. Please make sure it is accurate.<br><br>**Funded studies**<br>Enter a statement with the following details:<br>• Initials of the authors who received each award<br>• Grant numbers awarded to each author<br>• The full name of each funder<br>• URL of each funder website<br>• Did the sponsors or funders play any role in the study design, data collection and analysis, decision to publish, or preparation of the manuscript?<br><br>Did you receive funding for this work?	Yes
Please add funding details.<br>as follow-up to "**Financial Disclosure**<br><br>Enter a financial disclosure statement that describes the sources of funding for the work included in this submission. Review the submission guidelines for detailed requirements. View published research articles from *PLOS Neglected Tropical Diseases* for specific examples.<br><br>This statement is required for submission	The study was funded by Grand Challenges Canada and the Government of Canada

and **will appear in the published article** if the submission is accepted. Please make sure it is accurate.<br><br>**Funded studies**<br>Enter a statement with the following details:<br>• Initials of the authors who received each award<br>• Grant numbers awarded to each author<br>• The full name of each funder<br>• URL of each funder website<br>• Did the sponsors or funders play any role in the study design, data collection and analysis, decision to publish, or preparation of the manuscript?<br><br>Did you receive funding for this work?"	
Please select the country of your main research funder (please select carefully as in some cases this is used in fee calculation).<br>as follow-up to "**Financial Disclosure**<br><br>Enter a financial disclosure statement that describes the sources of funding for the work included in this submission. Review the submission guidelines for detailed requirements. View published research articles from *PLOS Neglected Tropical Diseases* for specific examples.<br><br>This statement is required for submission and **will appear in the published article** if the submission is accepted. Please make sure it is accurate.<br><br>**Funded studies**<br>Enter a statement with the following details:<br>• Initials of the authors who received each award<br>• Grant numbers awarded to each author<br>• The full name of each funder<br>• URL of each funder website<br>• Did the sponsors or funders play any role in the study design, data collection and analysis, decision to publish, or preparation of the manuscript?	CANADA - CA

Did you receive funding for this work?"	
**Competing Interests**<br><br>On behalf of all authors, disclose any competing interests that could be perceived to bias this work.<br><br>This statement will be typeset if the manuscript is accepted for publication.<br><br>Review the instructions link below and PLOS NTDs' competing interests policy to determine what information must be disclosed at submission.	No competing interest
**Data Availability**<br><br>Provide a **Data Availability Statement** in the box below. This statement should detail where the data used in this submission can be accessed. This statement will be typeset if the manuscript is accepted for publication.<br><br>Before publication, authors are required to make all data underlying their findings fully available, without restriction. Review our PLOS Data Policy page for detailed information on this policy. Instructions for writing your Data Availability statement can be accessed via the Instructions link below.	Relevant data and materials based on which conclusions were made are included in the manuscript. However, the corresponding author can be contacted if someone wants data from the study

Manuscript
Click here to access/download;Manuscript;Health workers knowledge about FGS.pdf

Breaking the silence of female genital schistosomiasis in Ghana's health system: A case of health workers within the FAST project

Margaret Gyapong[1], Maxwell Ayindenba Dalaba[1*], Mustapha Immurana[1], Alfred Kwesi Manyeh[1], Kazeem Arogundade[2], Julie Jacobson[3], Alison Krentel[2]*

[1]*Institute of Health Research, University of Health and Allied Sciences, Ho, Ghana*
[2]*Bruyère Research Institute, Ottawa, ON, Canada*
[3]*Bridges to Development, Vashon, WA, United States*

[*]Corresponding authors: Email: mgyapong@uhas.edu.gh (MG); mdalaba@uhas.edu.gh (MAD)

Abstract

Background: Female Genital Schistosomiasis (FGS) remains one a critical and neglected topics in Neglected Tropical Diseases (NTDs) and the health of women and girls worldwide. Health workers' knowledge of FGS is vital to the prevention and management of the disease. This study conducted implementation research to identify and address the FGS knowledge gap among health workers in Ghana.

Methods: This study was a 3-year (2020 -2022) implementation research applying a pragmatic uncontrolled quasi-experimental study design. The study involved a baseline assessment, an intervention phase involving the training of health workers about FGS and an endline assessment. A mixed-method approach was applied to data collection involving health workers in two schistosomiasis endemic districts and health workers across the country. NVIVO 12 and STATA 14 were used for qualitative and quantitative data analysis, respectively.

Results: Prior to the intervention, the level of awareness about FGS among health workers was less than 8%, and most participants only understood FGS as merely urogenital schistosomiasis in females.

In response to this gap, an FGS education intervention in the form of training of health workers, student nurses alongside the distribution of FGS educational materials were carried out. The intervention enhanced health workers' awareness of FGS to more than 61%, encompassing an enhanced understanding of the disease's signs and symptoms to more than 60% , as well as its management strategies.

Nevertheless, a key challenge remains in ensuring access to praziquantel, the primary medication for treating and preventing schistosomiasis.

Conclusions: The FGS intervention has improved health workers' awareness and understanding of FGS. However, there is still a need for expanded training of health workers as well as improvement in the access to praziquantel to facilitate FGS management. In addition,

a holistic strategy encompassing all stakeholders at the individual, community, and health-system levels is required to improve the general knowledge and management of FGS.

Keywords: Neglected Tropical Diseases, Schistosomiasis, Female Genital Schistosomiasis, Mass Drug Administration, Ghana.

Introduction

One of the most prevalent Neglected Tropical Diseases (NTDs) in Sub-Saharan Africa (SSA) is called schistosomiasis, bilharzia or "snail fever." Schistosomiasis is a parasitic disease that affects men, women and children living in tropical regions. Endemic in 78 countries, this disease affects more than 250 million people with approximately 90% of the disease burden found in Africa (1,2). Disease transmission occurs through contact with freshwater bodies where aquatic snails breed and releases the parasite that burrows through the skin in contact with water. Regular daily activities (washing, bathing, gathering water, swimming) put adults and children at continuous risk of infection. Current strategies to control schistosomiasis focus on preventive chemotherapy using donated praziquantel (1,2).

When a young girl or woman is infected with schistosomiasis, the worms mate producing millions of eggs every day inside her body. In the case of *S. haematobium*, the eggs are released into the genito-urinary tract embedding in the tissue causing inflammation that results in a granulomatous response around the eggs causing Female Genital Schistosomiasis (FGS)(3). Thus, FGS is a gynaecological disease caused by untreated urogenital schistosomiasis. FGS results in abdominal and pelvic pains, disorders of menstruation, pain during intercourse,

vaginal bleeding after intercourse, inflammation of reproductive organs, genital lesions, miscarriage, ectopic pregnancy and infertility (4).

Increasing evidence shows that a woman with FGS has a threefold increased risk of HIV infection when she becomes sexually active (5–7). Timely treatment can prevent the disease and reverse some of the associated biological risk factors even in chronic infection (8).

Nonetheless, FGS remains one of the most important neglected areas in women's and girls' health globally**.** An estimated 56 million women have FGS, nearly all in SSA (9). This infection is a greater concern for women and girls as their gender roles put them at a higher risk of being in contact with infested waters when completing household chores (10).

However, in Ghana, health workers' awareness of FGS and its associated consequences is lacking, and unfortunately even in places where the disease is endemic (11,12).

This study, therefore, conducted an implementation study to identify and address the FGS knowledge gap among health workers in Ghana. This involved a baseline assessment to examine the knowledge and understanding of FGS among health workers followed by an FGS intervention in the form of training and provision of educational materials to health workers.

Methods

Study Design

This was a 3-year (2020 -2022) implementation study applying a pragmatic uncontrolled quasi-experimental study design (pre and post-intervention) design. This paper is part of a bigger study called "FGS Accelerated Scale Together (FAST) Transition to scale project: improving women's health by reducing morbidity from female genital schistosomiasis in Ghana (FAST Project)". The main aim of the FAST project was to improve adolescent girls and women's health by reducing morbidity associated with FGS through preventive and curative efforts.

For this aspect of the paper, we report the study activities that involved heath workers in three phases of the study as described below:

Phase 1: Pre-intervention

This phase was a baseline assessment of the FGS knowledge gap among health workers in the country.

Firstly, interviews were held with health workers including directors of health and medical superintendents in two schistosomiasis-endemic districts in Ghana: North Tongu District and the Weija-Gbawe Municipality. The two districts were selected because they have several communities along the Volta Lake (the largest artificial reservoir in the world) and Wieja Dam (the second largest water reservoir in the country after the Volta reservoir), where the Ghana Health Service reports that schistosomiasis prevalence is at least 80% (13). Hence, these regions were chosen for the interviews to learn about the knowledge gaps among health workers, which can then be applied to other regions.

Secondly, we engaged relevant stakeholders such as Ghana Neglected Tropical Diseases Programme (NTDP) to identify policy gaps in FGS control. Following the discussions, the Ghana National FGS Committee was established to work towards helping to integrate FGS into routine health services.

Phase 2: Intervention

Phase two was the implementation of FGS intervention activities which was based on the baseline assessment. Four main activities were undertaken:

Online training of health workers

The FAST Package team, together with the Geneva Learning Foundation, Bridges to Development, conducted an online, interactive, peer-to-peer training on FGS targeting

healthcare professionals. The goals of the training were to train healthcare providers to integrate FGS into their clinical practice so that they can improve women's reproductive health by decreasing the burden of FGS and to encourage learners to co-create solutions within their local context to address FGS.

Of the 119 applicants who participated in the online FGS course/training held between May and June 2021, 62 successfully completed the entire session. The participants were a mix of doctors, obstetrics and gynecology specialists, nurses/midwives, physician assistants, health information officers, nursing educators, and public health professionals and lecturers from the northern, middle-belt, and southern geographical regions in Ghana.

As part of the training, participants developed feasible action plans to integrate FGS into their practice and establish and maintain awareness in their context. The action plans were designed to reflect actions that could be undertaken without additional budgetary support and within the learners' own domains of influence.

Face-to-face Subject Matter Experts (SME) training

To create a sustainable system of training healthcare providers within the health systems in Ghana, the FAST Package team, carried out a 3 day FGS training March 2022 to create a cohort of national FGS Subject Matter Experts. Individuals were first identified from the the online training program. These individuals had demonstrated intrinsic motivation to learn about FGS through their online participation. This resulted in a multidisciplinary range of training SMEs including obstetricians / gynecologists, doctors, nurses, midwifes and health information officers. This training had 30 participants.

After the training, participants were given 100 copies (5 copies each) of FGS booklets to distribute to their constituents to help disseminate FGS awareness and management. Expects in the field of FGS, home and abroad, collectively developed the FGS booklet to fit the context

of Ghana. The content of the FGS book included the causes, treatment and prevention of schistosomiasis and FGS (9). All participants (trainees and facilitators) were officially recognized as SMEs on FGS through the awarding of certificates upon completion of the training.

Training of frontline health workers on schistosomiasis at endemic districts

Training of frontline health workers was carried out in two schistosomiasis endemic districts (North Tongu and Weija-Gbawe Municipality). The total number of participants for the training was 116, of which 41 and 75 were from North Tongu and Weija-Gbawe Municipality respectively. After the training, 234 copies of the FGS booklets were distributed to health workers in North Tongu and 429 copies were distributed to health workers in Weija-Gbawe Municipality.

Face-to face training of health workers and students

A series of FGS training of health workers and students in the nursing, midwifery and medicine was carried out by the SMEs in their various continuants. For instance, within the period, series of lectures on FGS was delivered to over 300 nursing students, 100 midwifery students and 100 medicine students at UHAS, Ho in the Volta Region of Ghana. In addition, lectures on FGS were held for over 600 nursing and midwifery students at Nursing and Midwifery training college (NMTC), Berekum in the Eastern Region of Ghana.

Further, FGS presentations/education were made to about 700 health workers in the eastern region. The participants included: District directors of health, SHEP coordinators, nurses and NTD coordinators.

The Ghana National FGS Committee in collaboration with the Ghana NTDP organised schistosomiasis and FGS face-to-face awareness training for health workers in 15 districts in

the Volta, Western and Central regions of Ghana. Over 600 health workers were involved in the training on FGS.

Phase 3: Post-intervention

Phase three was the evaluation of the intervention. A cross-sectional mixed method approach was applied in data collection involving In-Depth Interviews (IDIs) with health workers as well as quantitative data of health workers in a pre- and post-training assessment format.

Data Collection, Management and Analysis

Graduate-level Research Assistants (RAs) who were trained for five days were used to collect the qualitative data. Interview guides were pretested before the actual data collection. The interviews were face-to-face, and the participants were purposively sampled to include Doctors, Nurses and Midwives based on their involvement in Obstetrics and gynaecology activities. A total of 4 IDIs and 16 IDIs were conducted at baseline and endline respectively. All qualitative interviews (IDIs) were audio recorded and later transcribed. The transcripts were reviewed for accuracy and completeness. Guided by the objectives of the study and the themes from the transcripts, a codebook was developed. Thematic analysis was carried out using NVIVO 12 software to interpret themes or patterns in the data pertinent to the study aims.

In addition, quantitative data were collected from 116 training participants in the form of pre and post-training evaluations to determine the changes in FGS knowledge among the health workers. This was done among health workers in the schistosomiasis endemic districts of North Tongu and Weija districts. The quantitative data were collected using a paper-based questionnaire and then entered STATA 14.0 for cleaning and analysis. The data were analysed using simple frequencies and percentages.

Ethical consideration

Ethical clearance for this study was obtained from the GHS Ethics Review Committee (GHS-ERC 003/04/21) and the University of Health and Allied Sciences Research Ethics Committee (UHAS-REC A.5 [4] 20-21). Written informed consent was obtained from all respondents in the study.

Results

This section presents quantitative results from pre- and post-training evaluations of health workers from North Tongu and Weija districts as well as online training evaluation across health workers in Ghana. The section also presents the qualitative findings from baseline and endline IDIs from North Tongu and Weija districts.

Socio-demographic characteristics

Table 1 presents the sociodemographic characteristics of frontline Health workers from the North Tongu and Weija districts who participated in the qualitative interviews (IDIs). The majority of the participants at the endline were females and the average age of the participants at baseline and endline was 34 and 48 years respectively.

Table 1 : Sociodemographic characteristics of IDI participants

	**Endline**		**Baseline**	
**Variable**	**Freq (16)**	**Percent (100%)**	**Freq (4)**	**Percent (100%)**
Sex				
Female	14	87.5	2	50
Male	2	12.5	2	50
Rank				
CHN	3	18.75	0	0
Medical Officer	3	18.75	2	50
Medical Superintendent	1	6.25	2	50
Midwife	2	12.5	0	0
Nurse	5	31.25	0	0
Physician Assistant	2	12.5	0	0

Education				
Degree	6	37.5	0	0
Diploma	7	43.75	0	0
Masters	3	18.75	4	100
**Average age**	34		48	
**Average number of years of services**	5		8	

Awareness about schistosomiasis and FGS

In the quantitative assessment in the two schistosomiasis endemic districts, the results showed that before FGS training, health workers' level of awareness (very aware) about FGS was less than 8% (North Tongu 7.3%; Weija 4.1%). Nonetheless, after the FGS training, more than 68% (North Tongu 68.3%; Weija 69.4%) of the participants mentioned that they were now very aware of FGS (Figure 1 and 2), representing more than 61% improvement in the level of level of awareness of FGS.

Similarly, in the qualitative assessment, the majority of the health workers who participated in the baseline and endline assessments indicated that they had heard of schistosomiasis. However, with regard to FGS, the knowledge level was low before the FGS training intervention. Furthermore, some of the health workers confessed that before the FGS training, they were not aware that women can get schistosomiasis and therefore they did not know about FGS prior to the training. They therefore acknowledged that the FGS intervention (FGS training and FGS booklet) has improved their competencies around FGS. These results suggest that the FGS training was effective in raising health workers' awareness of FGS.

*Figure 1: **Awareness about schistosomiasis and FGS (North Tongu)***

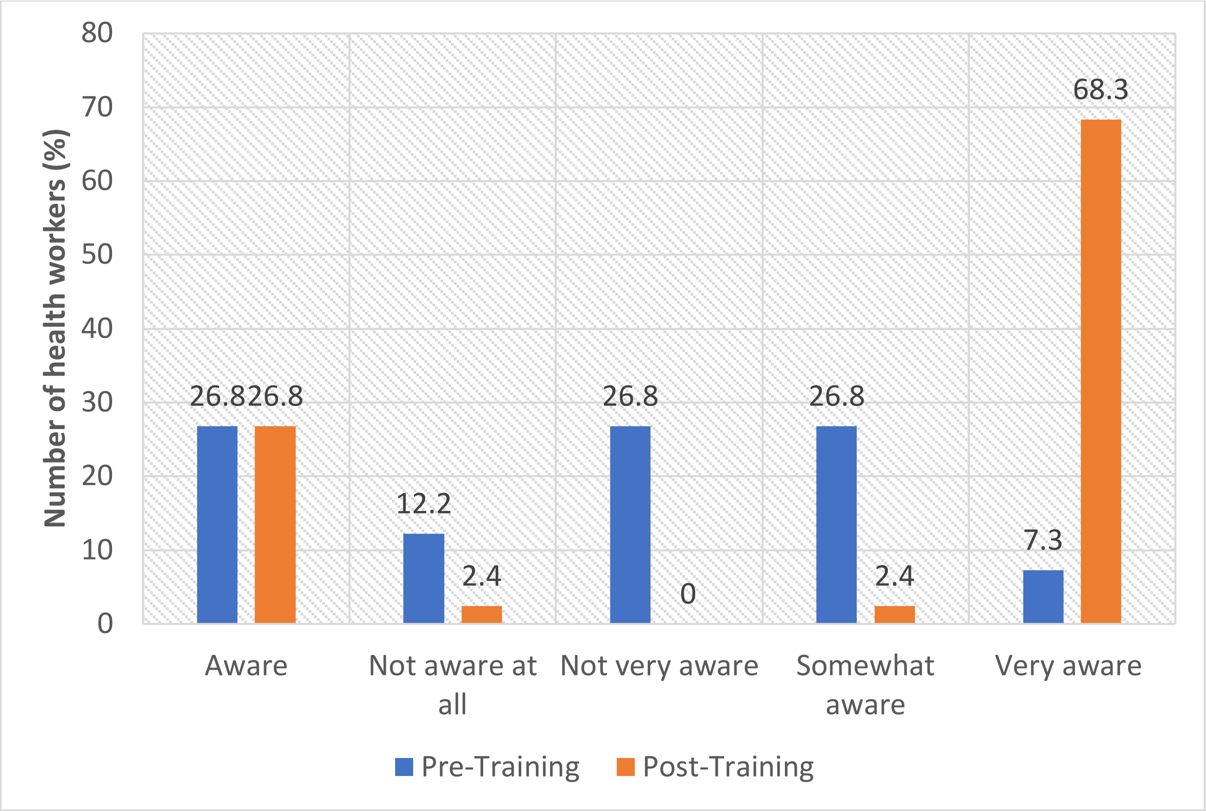

Figure 2 : ***Awareness about schistosomiasis and FGS (Weija)***

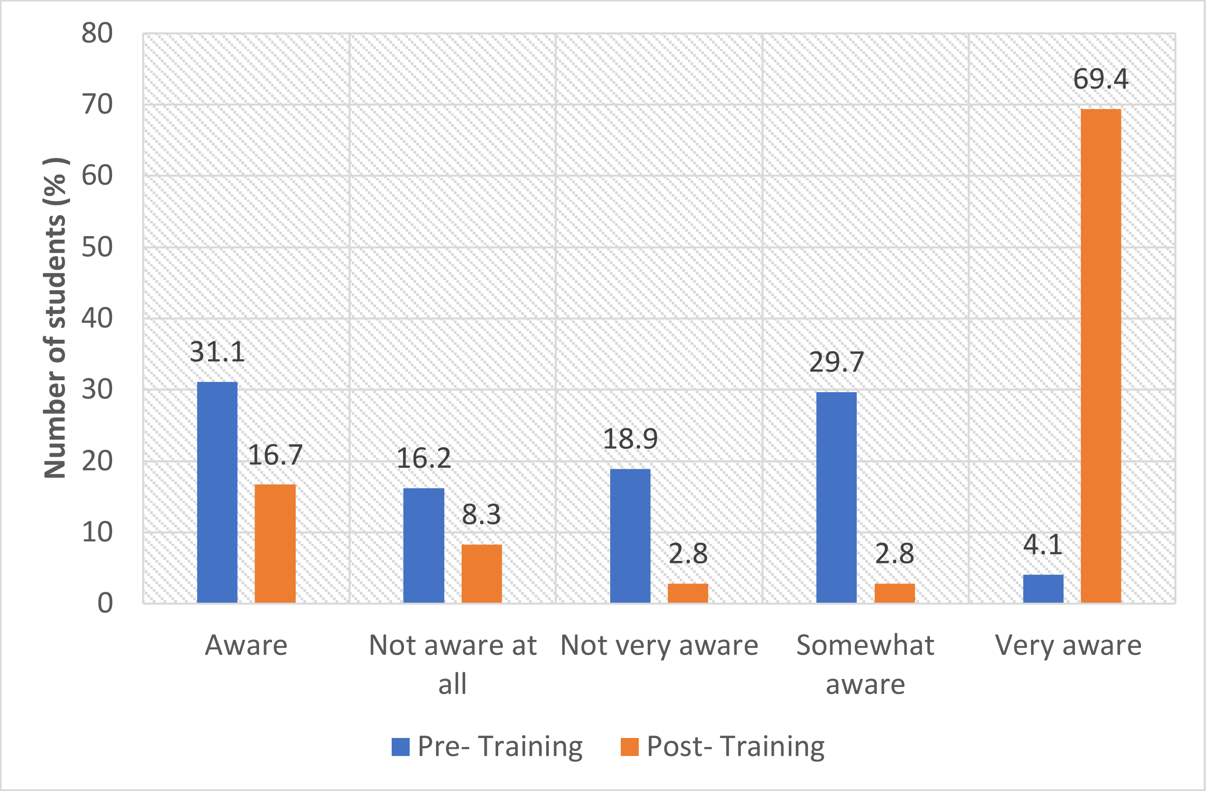

"Before this training, I knew nothing about FGS. What I knew about was the normal schistosomiasis (IDI-Health worker, North Tongu, Endline)"

"At first, I didn't know females get schistosomiasis. I knew about only the males. However, after the training, I am now aware of FGS…However, I did not get any FGS case after the training yet. In fact, I told one of my colleagues that I missed an FGS case because I encountered a lady before the FGS training and all the complaints she presented to me were related to FGS but because I was at that time not aware of FGS, I think a missed her. And there is no way I could trace her up (IDI-Health Worker, Weija, Endline).

Health workers knowledge in FGS diagnosis

Following the implementation of a FGS training intervention, there was a significant increase in healthcare workers' ability to diagnose FGS. In North Tongu, the percentage of health workers reporting confidence in FGS diagnosis rose from 26.8% to 97.6%, demonstrating an impressive 70.8% improvement. Similarly, in Weija, the proportion of confident healthcare workers increased from 35.4% to 95.8%, representing a notable 60.4% rise (Table 2). These findings illustrate the positive impact of training on healthcare workers' capacity to diagnose FGS effectively. This improved diagnostic ability will ultimately lead to earlier detection and treatment of FGS, contributing to improved patient outcomes and enhanced public health efforts.

Health workers knowledge in FGS Recording in DHIMS 2 System

The District Health Information Management System–2 (DHIMS 2) serves as the central database for capturing disease data within the healthcare system. However, prior to a recent training initiative, health workers lacked the necessary knowledge to identify and record

Female Genital Schistosomiasis (FGS) cases within the DHIMS 2 system. This resulted in an underreporting of FGS prevalence within the region. Following the implementation of a dedicated training program, a significant improvement in FGS data recording was observed. Health workers indicated that the training equipped them with the necessary knowledge and skills to effectively identify FGS cases and accurately record the information within the DHIMS 2 system. Thus, knowledge to identify and record FGS increased by 100%(Table 2).

Table 2 : ***Knowledge to diagnose, record and treat FGS***

	**North Tongu**		**Weija**	
	**Pre-Training**	**Post-Training**	**Pre-Training**	**Post-Training**
**Knowledge to diagnose FGS**				
No	30 (73.2)	1 (2.4)	42 (64.6)	3 (4.2)
Yes	11 (26.8)	40 (97.6)	23 (35.4)	69 (95.8)
Total	41 (100)	41 (100)	65 (100)	72 (100)
**Knowledge to treat FGS**				
No	30 (73.2)	2 (4.9)	48 (75)	0 (0)
Yes	11 (26.8)	39 (95.1)	16 (25)	71 (100)
Total	41 (100)	41 (100)	64 (100)	71 (100)
**Knowledge to refer FGS**				
No	24 (58.5)	0 (0)	26 (39.4)	4 (5.7)
Yes	17 (41.5)	41 (100)	40 (60.6)	66 (94.3)
Total	41 (100)	41 (100)	66 (100)	66 (100)
**Knowledge to record FGS**				
No	28 (68.3)	0 (0)	29 (44.6)	0 (0)

Yes	13 (31.7)	41 (100)	36 (55.4)	71 (100)
Total	41 (100)	41 (100)	65 (100)	71 (100)
**Knowledge to prevent FGS**				
No	22 (53.7)	0 (0)	24 (38.1)	0 (0)
Yes	19 (46.3)	41 (100)	39 (61.9)	71 (100)
Total	41 (100)	41 (100)	63 (100)	71 (100)

Knowledge of FGS from Online Training

Similarly, pre- and post-training evaluation of the FGS online training showed improvement in knowledge to diagnose, treat, record and prevent FGS (Figure 3). For instance, there was a 75.1% increase in knowledge to diagnose, amongst those who completed the course (Figure 3).

Figure 3: Knowledge of FGS from Online Training

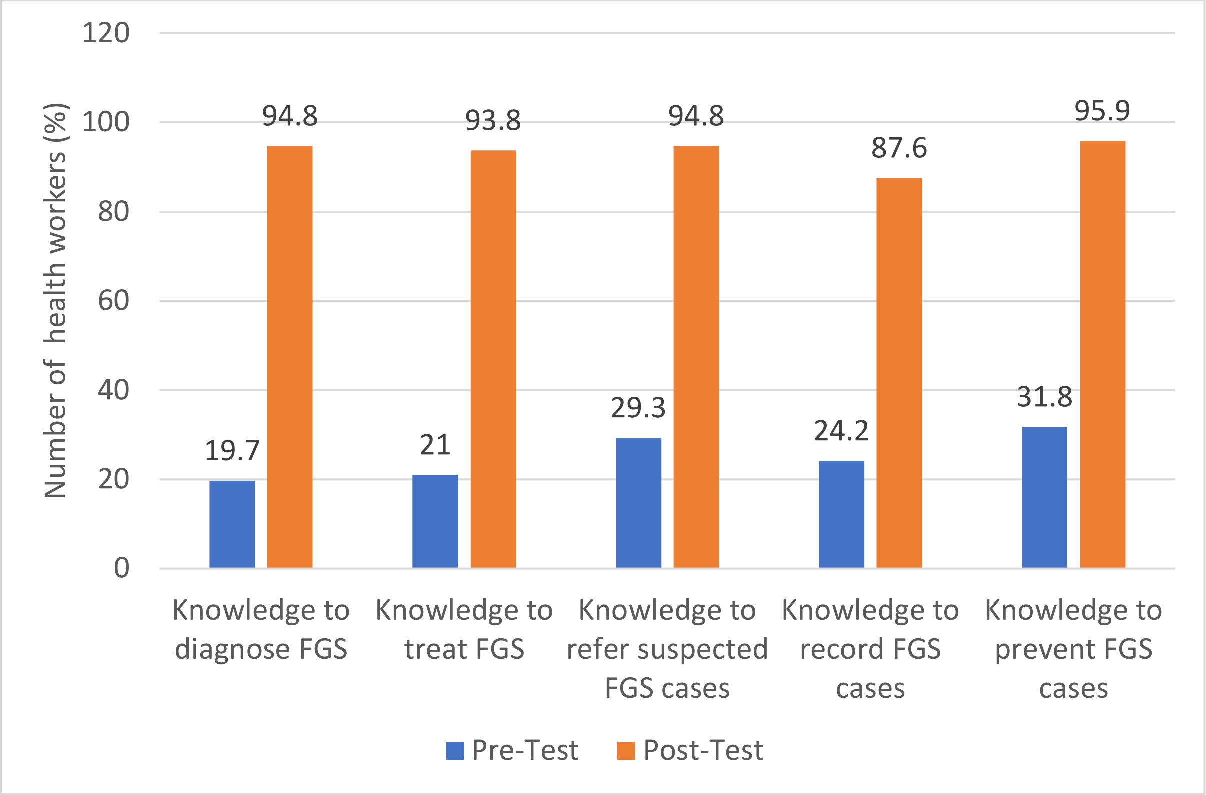

Scarcity of praziquantel hamper schistosomiasis and FGS treatment efforts

Healthcare workers reported encountering difficulty in obtaining praziquantel, often finding it unavailable at both health facilities and pharmacies. This scarcity forced them to adopt alternative approaches, such as occasionally resorting to antibiotics as a treatment option. In some cases, they would prescribe praziquantel to patients, leaving them to independently search for and purchase the medication at pharmacies, where its availability was uncertain.

"When they come to the health facility and we do not have praziquantel at the facility, I prescribe it to them to go buy from the pharmacy shop. Mostly we do not have praziquantel in the health facilities (IDI, Health Worker, Weija, Endline)"

"I think some of my colleagues sometimes give them antibiotics and painkillers if there is no praziquantel. Sometimes, after MDA, they give us the remaining praziquantel. If a patient comes and fortunately we have some, then we can give the praziquantel (IDI, Health Worker, North Tongu, Endline)"

FGS health seeking

Health care workers highlighted the occurrence of delayed care-seeking for schistosomiasis among women, often due to a lack of awareness about the disease and its potential progression to FGS. These women often remain unaware of their infection until they present for antenatal care (ANC) services. Notably, during ANC examinations, trained health workers can identify suspected cases of schistosomiasis/FGS. This was exemplified by a medical superintendent who recounted an instance where he implemented routine testing for schistosomiasis during ANC, leading to the identification of multiple cases.

"the very young ones do not know how they contracted it (schistosomiasis). ..like the routine tests that we do for the pregnant women, occasionally some of them test positive for schistosomiasis. I think we discharged a woman treated for FGS a month ago. I think we were just doing routine testing and then, schistosomiasis ova was present…"(IDI, medical superintendent, North Tongu).

The cost of treatment for schistosomiasis and FGS presents a barrier to seeking appropriate care, particularly for vulnerable populations. Healthcare workers reported that many community members either forgo treatment entirely or resort to low-cost alternatives due to financial constraints. These alternatives, such as herbal remedies or delaying treatment until mass drug administration (MDA) campaigns, may not be effective and can lead to further complications. The high cost of treatment can be attributed to the scarcity of praziquantel, the primary medication for schistosomiasis and FGS, at both health facilities. This scarcity often forces individuals to seek treatment at chemical shops, where prices are typically higher.

"Sometimes due to cost, parents do not treat the child when the child has schistosomiasis. They wait till it is time for MDA because, during that period, they can get it for free (IDI-Health worker, North Tongu, Baseline)"

Discussion

Gender roles expose females to schistosomiasis and FGS and therefore the need for strategic interventions, particularly in schistosomiasis endemic communities. The study sought to use

an implementation design to identify and address the FGS knowledge gap among health workers in Ghana.

The findings of the study revealed that the level of awareness of urogenital schistosomiasis as well as some common symptoms of schistosomiasis such as blood in the urine was high among health workers. However, it was observed that at the time of baseline assessment and before FGS training intervention, very little was known about FGS awareness, diagnosis and treatment. This finding is in tandem with previous studies in Tanzania and Nigeria that reported high knowledge about schistosomiasis among healthcare workers but limited knowledge about FGS (14,15). FGS is frequently given little attention in medical education and continues to be poorly understood by health workers, which commonly results in FGS being misdiagnosed or mistakenly diagnosed as an STI or cervical cancer and then given improper treatment (9). Traditionally, urogenital schistosomiasis has been primarily associated with male symptoms and this focus on male-specific manifestations has led to the neglect of FGS, which primarily affects women and often presents with less obvious symptoms (16,17).

Nonetheless, due to the study intervention, the level of awareness and knowledge on diagnosis and treatment of FGS among health workers was reported to have improved within the period, demonstrating the impact of the intervention. Continuous FGS education among health workers is very important in controlling FGS. The Ghana National FGS Committee mentioned that they would continue with the in-service training of nurses across the country and will also engage the Nurses and Midwifery Council concerning curriculum revision to incorporate FGS diagnosis and management. With these initiatives, FGS discussion will be reinforced at the school and practice levels. Improved competency about FGS among health workers will facilitate the prevention, diagnosis and treatment/management of FGS and therefore calls for more efforts to sensitize health workers about FGS beyond this study.

The result also showed that the intervention has improved knowledge and skills to effectively identify FGS cases and accurately record the information in the DHIMS 2 system. This will data capturing skills obtained will ultimately provide a more accurate picture of FGS prevalence, enabling targeted interventions and improved patient care.

Although praziquantel is the recommended medication for the treatment and prevention of schistosomiasis and FGS in Ghana (18–21), access and use of the medication is a challenge. Praziquantel is usually not available at health facilities for the treatment of schistosomiasis(15). This is not surprising given that praziquantel is a programmed medicine and it is only available during MDA and sparsely available in health facilities and pharmacies/drugs shops. In addition, those found at some of the pharmacies/drug shops are not affordable for community members. For instance, the price of a single 600mg tablet of Praziquantel is about GHc50 (USD5) and given that praziquantel is prescribed at a single dose of 40 mg per kilogram of body weight (9,18), if a child weighs 45kg, he or she will require as many as three tablets. The cost of treatment with praziquantel can be disastrous if a complete dose is considered, as single dose is more than three times the daily minimum wage (GH 14.88)(22) of a worker in Ghana. These findings highlight the need for continued efforts to address supply chain issues to ensure effective and accessible treatment for FGS patients (23).

Conclusion

There has been a lack of awareness and knowledge about Female Genital Schistosomiasis (FGS) among healthcare workers, leading to missed diagnoses and inadequate treatment. While the study intervention has improved some health workers awareness and the knowledge to diagnose and treat FGS, continued efforts are crucial to achieving greater impact.

We therefore recommend the following to expand FGS knowledge and awareness:

- Curriculum Integration: Incorporating FGS diagnosis and management into the training curriculum for healthcare workers at all levels is essential for equipping them with the necessary skills to identify and treat the disease.
- Equipped Facilities: Ensuring health facilities are adequately equipped with praziquantel, medical equipment, and supplies is crucial for effective diagnosis and treatment of FGS.
- Continued training and refresher courses: Providing ongoing training and refresher courses for healthcare workers helps ensure they stay up-to-date on the latest FGS information and treatment protocols.
- Public awareness campaigns: Raising public awareness about FGS symptoms, risk factors, and available treatment options is crucial for encouraging people to seek timely medical attention.
- Community Engagement: Engaging community leaders and health workers in raising awareness and promoting FGS prevention strategies can contribute significantly to reducing the disease burden.
- Collaboration and Partnerships: Fostering collaboration and partnerships between healthcare systems, research institutions, and NGOs can accelerate progress toward FGS elimination.

References

1. Loverde PT. Philip T. LoVerde 3 3.1. 2019;
2. WHO. Schistosomiasis Key Facts [Internet]. 2020 [cited 2020 Oct 21]. Available from: https://www.who.int/news-room/fact-sheets/detail/schistosomiasis
3. Orish VN, Morhe EKS, Azanu W, Alhassan RK, Gyapong M. The parasitology of female genital schistosomiasis. Curr Res Parasitol Vector Borne Dis. 2022 May 27;2:100093.

4. Yirenya-Tawiah DR, Ackumey MM, Bosompem KM. Knowledge and awareness of genital involvement and reproductive health consequences of urogenital schistosomiasis in endemic communities in Ghana: a cross-sectional study. Reproductive Health. 2016 Sep 21;13(1):117.

5. Brodish PH, Singh K. Association between Schistosoma haematobium exposure and human immunodeficiency virus infection among females in Mozambique. American Journal of Tropical Medicine and Hygiene. 2016;94(5):1040–4.

6. Downs JA, Dupnik KM, van Dam GJ, Urassa M, Lutonja P, Kornelis D, et al. Effects of schistosomiasis on susceptibility to HIV-1 infection and HIV-1 viral load at HIV-1 seroconversion: A nested case-control study. PLoS Neglected Tropical Diseases. 2017;11(9):1–15.

7. Kjetland EF, Ndhlovu PD, Gomo E, Mduluza T, Midzi N, Gwanzura L, et al. Association between genital schistosomiasis and HIV in rural Zimbabwean women. Aids. 2006;20(4):593–600.

8. Kleppa E, Ramsuran V, Zulu S, Karlsen GH, Bere A, Passmore JAS, et al. Effect of female genital schistosomiasis and anti-schistosomal treatment on monocytes, CD4+ T-Cells and CCR5 expression in the female genital tract. PLoS ONE. 2014;9(6):1–9.

9. Jacobson J, Pantelias A, Williamson M, Kjetland EF, Krentel A, Gyapong M, et al. Addressing a silent and neglected scourge in sexual and reproductive health in Sub-Saharan Africa by development of training competencies to improve prevention, diagnosis, and treatment of female genital schistosomiasis (FGS) for health workers. Reproductive Health. 2022 Jan 24;19(1):20.

10. UNAIDS. No more neglect Female genital schistosomiasis and HIV. 2019;44.

11. Kukula VA, MacPherson EE, Tsey IH, Stothard JR, Theobald S, Gyapong M. A major hurdle in the elimination of urogenital schistosomiasis revealed: Identifying key gaps in knowledge and understanding of female genital schistosomiasis within communities and local health workers. PLoS Negl Trop Dis. 2019 Mar;13(3):e0007207.

12. Mazani E, Taylor M, Kjetland EF, Ndhlovu PD. Knowledge and perceptions about schistosomiasis among primary school children and teachers in rural KwaZulu-Natal. S Afr J Infect Dis. 2020;35(1):126.

13. Ghana Health Service. Master plan for neglected tropical diseases programme, Ghana (2016-2020). [Internet]. 2020 [cited 2022 May 6]. Available from: https://espen.afro.who.int/system/files/content/resources/GHANA_NTD_Master_Plan_2016_2020.pdf

14. Mazigo HD, Samson A, Lambert VJ, Kosia AL, Ngoma DD, Murphy R, et al. Healthcare Workers' Low Knowledge of Female Genital Schistosomiasis and Proposed Interventions to Prevent, Control, and Manage the Disease in Zanzibar. Int J Public Health. 2022 Sep 15;67:1604767.

15. Aribodor OB, Mogaji HO, Surakat OA, Azugo NO, Jacob EC, Obikwelu EM, et al. Profiling the knowledge of female medical/para-medical students, and expertise of health

care professionals on female genital schistosomiasis in Anambra, South Eastern Nigeria. PLOS Neglected Tropical Diseases. 2023 Feb 16;17(2):e0011132.

16. Makia CM, Fesuh NB, Amabo EN, Gamba VA, Oluwole AS, Stothard R. Urogenital schistosomiasis (UGS) and female genital schistosomiasis (FGS) in Cameroon: an observational assessment of key reproductive health determinants of girls and women in the Matta Health Area. BMJ Open. 2023 Feb 1;13(2):e063392.

17. Mazigo HD, Samson A, Lambert VJ, Kosia AL, Ngoma DD, Murphy R, et al. "We know about schistosomiasis but we know nothing about FGS": A qualitative assessment of knowledge gaps about female genital schistosomiasis among communities living in Schistosoma haematobium endemic districts of Zanzibar and Northwestern Tanzania. PLOS Neglected Tropical Diseases. 2021 Sep 30;15(9):e0009789.

18. Darko SN, Hanson H, Twumasi-Ankrah S, Baffour-Awuah S, Adjei-Kusi P, Yar D, et al. Three monthly doses of 60 mg/kg praziquantel for Schistosoma haematobium infection is a safe and effective treatment regimen. BMC Infectious Diseases. 2020 May 6;20(1):323.

19. Dawaki S, Al-Mekhlafi HM, Ithoi I, Ibrahim J, Abdulsalam AM, Ahmed A, et al. The Menace of Schistosomiasis in Nigeria: Knowledge, Attitude, and Practices Regarding Schistosomiasis among Rural Communities in Kano State. PLOS ONE. 2015 Nov 25;10(11):e0143667.

20. Kokaliaris C, Garba A, Matuska M, Bronzan RN, Colley DG, Dorkenoo AM, et al. Effect of preventive chemotherapy with praziquantel on schistosomiasis among school-aged children in sub-Saharan Africa: a spatiotemporal modelling study. The Lancet Infectious Diseases. 2022 Jan 1;22(1):136–49.

21. Asuming-Brempong EK, Ayi I, Puije W van der, Gyan BA, Larbi IA, Ashong Y, et al. Increased ShTAL1 IgE responses post-Praziquantel treatment may be associated with a reduced risk to re-infection in a Ghanaian S. haematobium-endemic community. PLOS Neglected Tropical Diseases. 2022 Mar 9;16(3):e0010115.

22. Africa Pay. WageIndicator Foundation. 2023 [cited 2023 Apr 2]. Minimum Wages. Available from: https://africapay.org/ghana/salary/minimum-wages

23. Boakye G, Gyedu A, Stewart M, Donkor P, Mock C, Stewart B. Assessment of local supply chains and stock management practices for trauma care resources in Ghana: a comparative small sample cross-sectional study. BMC Health Services Research. 2021 Jan 13;21(1):66.
