# Supplementary material for: Breaking the silence of female genital schistosomiasis in Ghana’s health system: A case of health workers within the FAST project
Source: PLoS Negl Trop Dis. 2024 Sep 23;18(9):e0012443. doi: 10.1371/journal.pntd.0012443 (PMC11449374; doi:10.1371/journal.pntd.0012443)
Supplement: Attachment — Submitted filename: Cover letter_response to reviwers comments.docx [file pntd.0012443.s004.docx]

UNIVERSITY OF HEALTH AND ALLIED SCIENCES

Institute of Health Research

PMB 31

Ho Volta Region

Ghana

19^th^ June,2024

2

Tel: +233 362196193

email: [ihr@uhas.edu.gh](mailto:ihr@uhas.edu.gh)

My Ref:

Dear editorial board of PLOS Neglected Tropical Diseases,

We appreciate the opportunity to resubmit our manuscript entitled “**Breaking the silence of female genital schistosomiasis in Ghana’s health system: A case of health workers within the FAST project” for** publication in PLOS Neglected Tropical Diseases. We would like to thank the editors and reviewers for the useful comments. We have addressed all comments raised, with corresponding changes made to the manuscript where appropriate. Detailed responses to the comments are presented below:

**Reviewer One Comments**

Line 60: Introduction

**COMMENT**

**Please include some statements on the activities of the current schistosomiasis control programme in the area and indicate the shortcomings that have led to a lack of awareness about FGS and its poor management. Many endemic countries have school schistosomiasis control programmes. Is it the same in Ghana? If so, why should awareness be so low in a disease-endemic region? If the school programmes exist, are the girls included in the interventions? What is the involvement of the Ghana FGS Committee and the GNTP?**

**The absence of praziquantel in public health facilities in schistosomiasis regions is a critical observation that speaks to the disease programme's inadequacy. This needs to be elaborated on.**

RESPONSE

A statement has been added and it reads as:

“Schistosomiasis remains a major public health concern in Ghana, especially for residents of poor communities near freshwater sources. These areas often lack access to clean water and proper sanitation facilities. Despite the Ghana Health Service's efforts in conducting annual mass drug administration programmes and implementing some educational initiatives in schools, schistosomiasis remains prevalent (11). Moreover, there is a deficiency in educating health workers about schistosomiasis and FGS beyond the standard curriculum, leading to a lack of awareness among health workers about FGS and its associated consequences , even in places where the disease is endemic (12,13)”.

(Location of revision: Page 5, Line 105-111)

**COMMENT**

**Line 76: More complications of schistosomiasis could be included, especially urogenital cancers such as bladder.**

RESPONSE

This has been added

**COMMENT**

**Line 192; Ethical considerations**

**A study like this one that is likely to influence policy change would normally receive additional approvals from the Ministry of Health. If the authors could mention that, it adds credence to the paper. Also, include a statement that participants volunteered to respond to the study's requirements.**

RESPONSE

This has been revised accordingly. The Ghana Health Service is under the Ministry of Health and they provide ethical clearance for studies like this.

(Location of revision: Page 10, Line 244-247)

**COMMENT**

**Line 199-202: It would enrich the text (especially for international readers) to include a map indicating the location of the study districts where schistosomiasis is endemic in Ghana.**

RESPONSE

A map has been added

(Location of revision: Figure 1)

**COMMENT**

**Line 209: Table one**

1. **Consistency in decimal placement would improve the quality of the table. One decimal place for percentages is enough to convey the message.**
2. **CHN should be in full to enhance the understanding of the table contents and relate to the text.**

RESPONSE

This has been revise accordingly

(Location of revision: Page 10-11, Table 1)

**COMMENT**

**Line 226 Figure One**

1. **It is concerning that some health workers remained unaware of FGS even after training. Therefore, explanations should be given for this peculiar finding. Is it possible that health workers who did not undergo training, also became more aware about FGS through their peers?**

RESPONSE

This has been added to the discussion section and it reads as:

Furthermore, health workers who did not participate in the FGS intervention will gain awareness about FGS. This is because the training adopted a "trainer of trainers" approach, where participants received FGS materials to disseminate among their colleagues, thus enhancing overall understanding of FGS.

(Location of revision: Page 17, Line 439-442)

**COMMENT**

**Line 347: The sentence is unclear. This could be due to a typo or an omission that caused an unintended grammatical error. Please correct that sentence.**

RESPONSE

This has been revised and now reads:

“Understanding diseases is crucial for accurately capturing data into DHIMS 2 to assess their prevalence and to make policy decisions(19). The study's findings indicated that the intervention enhanced both knowledge and skills, enabling better identification and precise documentation of FGS cases in the DHIMS 2 system. These acquired data-capturing abilities will lead to a more precise understanding of FGS prevalence, facilitating targeted interventions and enhanced patient care”

(Location of revision: Page 17, Line 451-456)

**COMMENT**

**Lines 361-362: Treatment of schistosomiasis in females and males, including children. Young children who normally accompany females during their daily chores in infested water bodies are also at risk of infection, and mentioning that fact in the recommended actions would add more information and enrich the paper.**

**Lines 376-378: Inclusion of statements on advocacy for a comprehensive schistosomiasis control programme that is holistic and includes boys and girls (FGS), schools and the community**

RESPONSE

This has been added as:

- Comprehensive approach to FGS: Holistic approach to FGS: FGS elimination should be achieved through a comprehensive and holistic programme package covering all community members (males, females and children) at risk of contracting the disease.

(Location of revision: Page 20, Line 536-538)

**COMMENT**

**Lines 385-387: As stated above, FGS elimination should be achieved through a comprehensive and holistic programme package covering all community members (males and females) at risk of contracting the disease.**

RESPONSE

This has been added as:

- Comprehensive approach to FGS: Holistic approach to FGS: FGS elimination should be achieved through a comprehensive and holistic programme package covering all community members (males, females and children) at risk of contracting the disease.

(Location of revision: Page 20, Line 536-538)

**REVIEWER 2**

**COMMENT**

**Key words should be expanded to include knowledge gap,Health workers, Fast Project and remove mass Drug Administration. The focus of the study was to identify and address, hence inclusion amongst the key words**

RESPONSE

This has been revised as:

**Keywords**: Schistosomiasis, Female Genital Schistosomiasis, Health workers, knowledge gap, FAST Project, Ghana.

(Location of revision: Page 3, Line 54-55)

**COMMENT**

**Replace this with the disease**

RESPONSE

This has been replaced with “ the disease”

(Location of revision: Page 2, Line 49)

**COMMENT**

**Replace study with the team or investigators**

RESPONSE

This has been replaced by “ the investigators”

(Location of revision: Page 5, Line 114)

**COMMENT**

**The health workers trained on the FGS FAST Project, do they have the authority to integrate this training into their clinical practice without a formal approval from the management and policy makers?**

RESPONSE

We have revised the sentence to make it clearer. It reads:

The training aimed to equip healthcare providers with essential knowledge about FGS, enabling them to integrate FGS into their clinical practice. This will help to improve women’s reproductive health by decreasing the burden of FGS. Additionally, the training seeks to encourage learners to co-create solutions within their local context to address FGS.

(Location of revision: Page 6-7, Line 158-166)

**COMMENT**

**Is it medical or medicine students?**

RESPONSE

This has been changed to “medical students”

(Location of revision: Page 7, Line 170)

**COMMENT**

**Title of figures should be down except if is the format recommended by the PLOS NTDs.**

RESPONSE

This has been moved down

**COMMENT**

**Is it chemical or chemist shops?**

RESPONSE

This has been changes to “chemist shops”

(Location of revision: Page 16, Line 412)

**COMMENT**

**You need to recast the opening statement.**

RESPONSE

This has been revised to:

Understanding diseases is crucial for accurately capturing data to assess their prevalence. The study's findings indicated that the intervention enhanced both knowledge and skills, enabling better identification and precise documentation of FGS cases in the DHIMS 2 system. These acquired data-capturing abilities will lead to a more precise understanding of FGS prevalence, facilitating targeted interventions and enhanced patient care.

(Location of revision: Page 17, Line 452-457)

**COMMENT**

**The implication of this is that the Ghana NTDs Programme is not complying with the WHO new guidelines that PQZ should be available at the health facilities for treatment of all infected persons irrespective of age, including infected pregnant women after the first trimester, lactating mothers and child under two years of age. There is need for the Ghana health service to enhance collaboration and vigorously pursue integration of schistosomiaisis management into the health system.**

RESPONSE

This section has been revised accordingly and now reads:

However, this contradicts the latest WHO guidelines, which advocate for praziquantel's availability at health facilities for treating all infected individuals, regardless of age, including pregnant women beyond the first trimester, lactating mothers, and children under two years old (25) . This underscores the necessity for the Ghana Health Service to intensify collaboration and actively pursue the integration of schistosomiasis management into the healthcare system.

(Location of revision: Page 18, Line 4735-479)

**COMMENT**

**The analysis presented matched the analysis plan. The results were clearly presented with figures and tables. However the authors should have carried comparative analysis of the different categories of health workers in respect of their level of knowledge in identification, diagnosis, and management of FGS pre and after the training. Further statistical analysis may highlight whether there statistical significance amongst them. This will provide further insights in targeting and addressing the identified gaps**

RESPONSE

All participants in the training were nurses and midwives, reflecting their primary role in obstetrics and gynecology. As they share the same professional level, a comparative analysis by profession was not performed.

This has been made clearer in the manuscript and also been acknowledged in the study limitations.

**COMMENTS**

**The conclusions supported the data presented however the limitations of the study were not highlighted**.

RESPONSE

Limitations section has been added as:

**“Limitations of the study**

One potential limitation is the presence of selection bias. Training frontline health workers on schistosomiasis in endemic districts was restricted to nurses and midwives due to their involvement in obstetrics and gynecology. While this choice aligns with the focus on FGS diagnosis, it excludes other healthcare providers, such as general practitioners and pediatricians, who may also encounter FGS patients. However, we assumed that these excluded professionals possess sufficient knowledge about FGS due to their higher professional status.

Another limitation relates to the sample size. The limited sample may not accurately reflect the overall knowledge gap among all healthcare workers. Nevertheless, we do not anticipate variations in knowledge levels of health workers among similar settings, as they likely underwent comparable professional training.

Additionally, there is limited evidence regarding the intervention's impact. Although the study effectively assessed changes in awareness and knowledge, it did not directly evaluate its effect on actual diagnosis and treatment practices for FGS patients. Nonetheless, we remain hopeful that participants will translate their newfound knowledge into practical application”

(Location of revision: Page 18-19, Line 490-511)

The authors will be happy to clarify further issues that the reviewers may raise. However, we hope that the concerns raised are adequately addressed.

Accompanying this response letter is a revised version of our manuscript.

**Sincerely,**

Maxwell Dalaba (on behalf of the authors)
