# Supplementary material for: Breaking the silence of female genital schistosomiasis in Ghana’s health system: A case of health workers within the FAST project
Source: PLoS Negl Trop Dis. 2024 Sep 23;18(9):e0012443. doi: 10.1371/journal.pntd.0012443 (PMC11449374; doi:10.1371/journal.pntd.0012443)
Supplement: Attachment — Submitted filename: Cover letter_response to reviwers comments_01_08_2024.docx [file pntd.0012443.s006.docx]

UNIVERSITY OF HEALTH AND ALLIED SCIENCES

Institute of Health Research

PMB 31

Ho Volta Region

Ghana

01^2t^ August,2024

2

Tel: +233 362196193

email: [ihr@uhas.edu.gh](mailto:ihr@uhas.edu.gh)

My Ref:

Dear editorial board of PLOS Neglected Tropical Diseases,

We appreciate the opportunity to resubmit our manuscript entitled “**Breaking the silence of female genital schistosomiasis in Ghana’s health system: A case of health workers within the FAST project”** for publication in PLOS Neglected Tropical Diseases. We would like to thank the editors and reviewers for the useful comments. We have addressed all comments raised, with corresponding changes made to the manuscript where appropriate. Detailed responses to the comments are presented below:

**Reviewer One Comments**

No comments from the reviewer

**Reviewer two Comments**

**COMMENT: The authors should incorporate secondary statistical analysis to support the study findings.**

**RESPONSE**

Secondary data on FGS from DHIMS for the period between 2021 and 2023 has been added.

*“DHIMS 2 data analysis revealed that FGS case reporting is ongoing. In 2021, 2022, and 2023, a total of 329, 147, and 69 FGS cases, respectively, were recorded (Table 3).*

**Table 3: Records of Schistosomiasis and FGS in the DHIMS 2 data.**

| ***Region*** | ***Schistosomiasis (2022)*** | ***Schistosomiasis (2023)*** | ***Female Genital Schistosomiasis (2022)*** | ***Female Genital Schistosomiasis (2023)*** |
| --- | --- | --- | --- | --- |
| *Ahafo* | *20* | *4* | *0* | *0* |
| *Ashanti* | *548* | *294* | *14* | *11* |
| *Bono* | *15* | *110* | *0* | *0* |
| *Bono East* | *259* | *384* | *13* | *17* |
| *Central* | *249* | *222* | *6* | *3* |
| *Eastern* | *543* | *473* |  | *12* |
| *Greater Accra* | *375* | *525* | *91* | *7* |
| *North East* | *5* | *2* | *0* | *0* |
| *Northern* | *26* | *37* | *0* | *4* |
| *Oti* | *99* | *70* | *0* | *0* |
| *Savannah* | *44* | *85* | *0* | *0* |
| *Upper East* | *82* | *135* | *0* | *3* |
| *Upper West* | *79* | *46* | *1* | *1* |
| *Volta* | *175* | *267* | *13* | *9* |
| *Western* | *191* | *150* | *9* | *1* |
| *Western North* | *60* | *50* | *0* | *1* |
| ***Ghana*** | ***2770*** | ***2854*** | ***147*** | ***69*** |

*“*

(Location of review: Page 14, line 315-318)

**COMMENT: The limitations were not clearly stated. The public health relevance were stated.**

RESPONSE: The limitation sections has been revised and now reads as:

***“Selection Bias****: Training frontline health workers on schistosomiasis in endemic districts was restricted to nurses and midwives due to their involvement in obstetrics and gynecology. While this choice aligns with the focus on FGS diagnosis, it excludes other healthcare providers, such as general practitioners and pediatricians, who may also encounter FGS patients. However, we assumed that these excluded professionals possess sufficient knowledge about FGS due to their higher professional status and therefore will not affect the study findings.*

***Limited Sample Size****: The relatively small number of trained health workers may restrict the study's impact to a limited population, potentially affecting its representativeness at the national level. To mitigate this, trained personnel were encouraged to disseminate their knowledge to colleagues.*

***Outcome Measurement****: While the study effectively assessed changes in awareness and knowledge, it did not directly measure the impact on FGS diagnosis and treatment practices. This limits our ability to fully evaluate the intervention's effectiveness in improving patient care. Nevertheless, it is anticipated that participants will apply their newfound knowledge in practice”*

(Location of review: Page 19, line 429-443)

The authors will be happy to clarify further issues that the reviewers may raise. However, we hope that the concerns raised are adequately addressed.

Accompanying this response letter is a revised version of our manuscript.

**Sincerely,**

Maxwell Dalaba (on behalf of the authors)
