# Supplementary material for: Breaking the silence of female genital schistosomiasis in Ghana’s health system: A case of health workers within the FAST project
Source: PLoS Negl Trop Dis. 2024 Sep 23;18(9):e0012443. doi: 10.1371/journal.pntd.0012443 (PMC11449374; doi:10.1371/journal.pntd.0012443)
Supplement: Peer review history

# Peer review: Decision Letter 0

**DOI:** 10.1371/journal.pntd.0012443.r001

**Authors:** Guilherme L Werneck, Elsio A Wunder Jr

8 Jun 2024

Dear Dr. Dalaba,

Thank you very much for submitting your manuscript "Breaking the silence of female genital schistosomiasis in Ghana’s health system: A case of health workers within the FAST project" for consideration at PLOS Neglected Tropical Diseases. As with all papers reviewed by the journal, your manuscript was reviewed by members of the editorial board and by several independent reviewers. In light of the reviews (below this email), we would like to invite the resubmission of a significantly-revised version that takes into account the reviewers' comments. 

The reviewers positively evaluated the manuscript and indicated minor revisions. However, they also made necessary suggestions and recommendations that might require a significant effort to review the manuscript. Please note their comments not only in the evaluation form but also in the attached material. In particular, reviewer #2 considered critical some "comparative analysis of the different categories of health workers in respect of their level of knowledge in identification, diagnosis, and management of FGS pre and after the training." Please make sure to conduct such an analysis or present some solid argument for not doing so.

We cannot make any decision about publication until we have seen the revised manuscript and your response to the reviewers' comments. Your revised manuscript is also likely to be sent to reviewers for further evaluation.

When you are ready to resubmit, please upload the following:

[1] A letter containing a detailed list of your responses to the review comments and a description of the changes you have made in the manuscript. Please note while forming your response, if your article is accepted, you may have the opportunity to make the peer review history publicly available. The record will include editor decision letters (with reviews) and your responses to reviewer comments. If eligible, we will contact you to opt in or out.

[2] Two versions of the revised manuscript: one with either highlights or tracked changes denoting where the text has been changed; the other a clean version (uploaded as the manuscript file). 

Important additional instructions are given below your reviewer comments.

Please prepare and submit your revised manuscript within 60 days. If you anticipate any delay, please let us know the expected resubmission date by replying to this email. Please note that revised manuscripts received after the 60-day due date may require evaluation and peer review similar to newly submitted manuscripts.

Thank you again for your submission. We hope that our editorial process has been constructive so far, and we welcome your feedback at any time. Please don't hesitate to contact us if you have any questions or comments.

Sincerely,

Guilherme L Werneck

Academic Editor

PLOS Neglected Tropical Diseases

Elsio Wunder Jr

Section Editor

PLOS Neglected Tropical Diseases

***********************

The reviewers positively evaluated the manuscript and indicated minor revisions. However, they also made necessary suggestions and recommendations that might require a significant effort to review the manuscript. Please note their comments not only in the evaluation form but also in the attached material. In particular, reviewer #2 considered critical some "comparative analysis of the different categories of health workers in respect of their level of knowledge in identification, diagnosis, and management of FGS pre and after the training." Please make sure to conduct such an analysis or present some solid argument for not doing so.

Reviewer's Responses to Questions

**Key Review Criteria Required for Acceptance?**

As you describe the new analyses required for acceptance, please consider the following:

**Methods**

-Are the objectives of the study clearly articulated with a clear testable hypothesis stated?

-Is the study design appropriate to address the stated objectives?

-Is the population clearly described and appropriate for the hypothesis being tested?

-Is the sample size sufficient to ensure adequate power to address the hypothesis being tested?

-Were correct statistical analysis used to support conclusions?

-Are there concerns about ethical or regulatory requirements being met?

Reviewer #1: Comments about the method are attached. Overall, the design, objectives and methods are satisfactory

Ethical consideration is addressed in the attachment. A request is made to include approvals from health authorities. The authors should also indicate that the study participants did so voluntarily .

Otherwise, these have been done well

Reviewer #2: The objective of the study was clearly stated. The study design is suitable with quasi-experimental design and mixed method approach deployed. The study population was clearly described and appropriate for the study design. The sample size is quite sufficient. Correct statistical analysis was used to support the data. The NVIVO 12 and STATA 14 used for the qualitative and quantitative analysis was adequate. 

No concerns.

--------------------

**Results**

-Does the analysis presented match the analysis plan?

-Are the results clearly and completely presented?

-Are the figures (Tables, Images) of sufficient quality for clarity?

Reviewer #1: The analysis is in tandem with the objectives.

A request to include a map is being made. Correction of decimal places is needed.

With these minor corrections the above are completed.

Reviewer #2: The analysis presented matched the analysis plan. The results were clearly presented with figures and tables. However the authors should have carried comparative analysis of the different categories of health workers in respect of their level of knowledge in identification, diagnosis, and management of FGS pre and after the training. Further statistical analysis may highlight whether there statistical significance amongst them. This will provide further insights in targeting and addressing the identified gaps.

--------------------

**Conclusions**

-Are the conclusions supported by the data presented?

-Are the limitations of analysis clearly described?

-Do the authors discuss how these data can be helpful to advance our understanding of the topic under study?

-Is public health relevance addressed?

Reviewer #1: Comments are indicated in the attached text. Overall, this is also adequate

Reviewer #2: The conclusions supported the data presented however the limitations of the study were not highlighted. The authors described how the data will enhance our understanding of the topic under study. The public health relevance was addressed with recommendations made.

--------------------

**Editorial and Data Presentation Modifications?**

Use this section for editorial suggestions as well as relatively minor modifications of existing data that would enhance clarity. If the only modifications needed are minor and/or editorial, you may wish to recommend “Minor Revision” or “Accept”. 

Reviewer #1: The suggestions are in the attached review comments

Reviewer #2: Accept the manuscript after minor modification. However the authors should have carried comparative analysis of the different categories of health workers in respect of their level of knowledge in identification, diagnosis, and management of FGS pre and after the training. Further statistical analysis may highlight whether there are statistical significance amongst them. This will provide more insights in addressing the identified gaps.

--------------------

**Summary and General Comments**

Use this section to provide overall comments, discuss strengths/weaknesses of the study, novelty, significance, general execution and scholarship. You may also include additional comments for the author, including concerns about dual publication, research ethics, or publication ethics. If requesting major revision, please articulate the new experiments that are needed.

Reviewer #1: This is basically a straightforward paper which is adequately presented. With those minor revisions the paper should be accepted

Reviewer #2: The manuscript was well written. The writing and language deployed in developing the manuscript is clear, with figures and tables presented to support the findings.

--------------------

PLOS authors have the option to publish the peer review history of their article (what does this mean?). If published, this will include your full peer review and any attached files.

If you choose “no”, your identity will remain anonymous but your review may still be made public.

**Do you want your identity to be public for this peer review?** For information about this choice, including consent withdrawal, please see our Privacy Policy.

Reviewer #1: Yes: Professor Modest Mulenga

Reviewer #2: Yes: Obiageli Josephine Nebe

Figure Files:

While revising your submission, please upload your figure files to the Preflight Analysis and Conversion Engine (PACE) digital diagnostic tool, https://pacev2.apexcovantage.com. PACE helps ensure that figures meet PLOS requirements. To use PACE, you must first register as a user. Then, login and navigate to the UPLOAD tab, where you will find detailed instructions on how to use the tool. If you encounter any issues or have any questions when using PACE, please email us at figures@plos.org.

Data Requirements:

Please note that, as a condition of publication, PLOS' data policy requires that you make available all data used to draw the conclusions outlined in your manuscript. Data must be deposited in an appropriate repository, included within the body of the manuscript, or uploaded as supporting information. This includes all numerical values that were used to generate graphs, histograms etc.. For an example see here: http://www.plosbiology.org/article/info%3Adoi%2F10.1371%2Fjournal.pbio.1001908#s5.

Reproducibility:

To enhance the reproducibility of your results, we recommend that you deposit your laboratory protocols in protocols.io, where a protocol can be assigned its own identifier (DOI) such that it can be cited independently in the future. Additionally, PLOS ONE offers an option to publish peer-reviewed clinical study protocols. Read more information on sharing protocols at https://plos.org/protocols?utm_medium=editorial-email&utm_source=authorletters&utm_campaign=protocols

Attachment Submitted filename: PNTD-D-24-00259_reviewer Ghana Project Reviewer One.pdf

Attachment Submitted filename: PNTD-D-24-00259 FGS manuscript in Ghana.pdf

# Author comment: Author response to Decision Letter 0

**DOI:** 10.1371/journal.pntd.0012443.r002

1 Jul 2024

Attachment Submitted filename: Cover letter_response to reviwers comments.docx

# Peer review: Decision Letter 1

**DOI:** 10.1371/journal.pntd.0012443.r003

**Authors:** Guilherme L Werneck

15 Jul 2024

Dear Dr. Dalaba,

Thank you very much for submitting your manuscript "Breaking the silence of female genital schistosomiasis in Ghana’s health system: A case of health workers within the FAST project" for consideration at PLOS Neglected Tropical Diseases. As with all papers reviewed by the journal, your manuscript was reviewed by members of the editorial board and by several independent reviewers. The reviewers appreciated the attention to an important topic. Based on the reviews, we are likely to accept this manuscript for publication, providing that you modify the manuscript according to the review recommendations. 

Both reviewers considered that the revision addressed most of the points raised in the first round of review. However, Reviewer #2 recorded some minor issues in the review report and in the manuscript that the authors should address. Not all recommendations are obligatory, but authors should provide reasoned answers to the issues raised.

Please prepare and submit your revised manuscript within 30 days. If you anticipate any delay, please let us know the expected resubmission date by replying to this email. 

When you are ready to resubmit, please upload the following:

[1] A letter containing a detailed list of your responses to all review comments, and a description of the changes you have made in the manuscript. 

Please note while forming your response, if your article is accepted, you may have the opportunity to make the peer review history publicly available. The record will include editor decision letters (with reviews) and your responses to reviewer comments. If eligible, we will contact you to opt in or out

[2] Two versions of the revised manuscript: one with either highlights or tracked changes denoting where the text has been changed; the other a clean version (uploaded as the manuscript file).

Important additional instructions are given below your reviewer comments. 

Thank you again for your submission to our journal. We hope that our editorial process has been constructive so far, and we welcome your feedback at any time. Please don't hesitate to contact us if you have any questions or comments.

Sincerely,

Guilherme L Werneck

Section Editor

PLOS Neglected Tropical Diseases

Elsio Wunder Jr

Section Editor

PLOS Neglected Tropical Diseases

***********************

Both reviewers considered that the revision addressed most of the points raised in the first round of review. However, Reviewer #2 recorded some minor issues in the review report and in the manuscript that the authors should address. Not all recommendations are obligatory, but authors should provide reasoned answers to the issues raised.

Reviewer's Responses to Questions

**Key Review Criteria Required for Acceptance?**

As you describe the new analyses required for acceptance, please consider the following:

**Methods**

-Are the objectives of the study clearly articulated with a clear testable hypothesis stated?

-Is the study design appropriate to address the stated objectives?

-Is the population clearly described and appropriate for the hypothesis being tested?

-Is the sample size sufficient to ensure adequate power to address the hypothesis being tested?

-Were correct statistical analysis used to support conclusions?

-Are there concerns about ethical or regulatory requirements being met?

Reviewer #1: This has been done

Reviewer #2: The objectives were clearly stated, with appropriate study design. The study population was well described, with sufficient sample size.

The authors should incorporate secondary statistical analysis to support the study findings.

No concerns.

--------------------

**Results**

-Does the analysis presented match the analysis plan?

-Are the results clearly and completely presented?

-Are the figures (Tables, Images) of sufficient quality for clarity?

Reviewer #1: This has been done

Reviewer #2: The results match the analysis. The figures are of sufficient quality.

--------------------

**Conclusions**

-Are the conclusions supported by the data presented?

-Are the limitations of analysis clearly described?

-Do the authors discuss how these data can be helpful to advance our understanding of the topic under study?

-Is public health relevance addressed?

Reviewer #1: This has been done

Reviewer #2: They are supported by the data presented. The limitations were not clearly stated. The public health relevance were stated.

--------------------

**Editorial and Data Presentation Modifications?**

Use this section for editorial suggestions as well as relatively minor modifications of existing data that would enhance clarity. If the only modifications needed are minor and/or editorial, you may wish to recommend “Minor Revision” or “Accept”. 

Reviewer #1: Accept

Reviewer #2: Minor revision required. The authors should have done secondary analysis to support the findings of the study.

--------------------

**Summary and General Comments**

Use this section to provide overall comments, discuss strengths/weaknesses of the study, novelty, significance, general execution and scholarship. You may also include additional comments for the author, including concerns about dual publication, research ethics, or publication ethics. If requesting major revision, please articulate the new experiments that are needed.

Reviewer #1: As indicated in the attached file, only minor typos are included. The paper is straightforward and ready for publication

Reviewer #2: The research findings highlighted the public health relevance of the study but limitations of the study was not stated.

Secondary analysis should also be carried out to support the findings. 

The authors should clarify and address the comments made in the manuscript.

--------------------

PLOS authors have the option to publish the peer review history of their article (what does this mean?). If published, this will include your full peer review and any attached files.

If you choose “no”, your identity will remain anonymous but your review may still be made public.

**Do you want your identity to be public for this peer review?** For information about this choice, including consent withdrawal, please see our Privacy Policy.

Reviewer #1: No

Reviewer #2: Yes: Obiageli Josephine Nebe

Figure Files:

While revising your submission, please upload your figure files to the Preflight Analysis and Conversion Engine (PACE) digital diagnostic tool, https://pacev2.apexcovantage.com. PACE helps ensure that figures meet PLOS requirements. To use PACE, you must first register as a user. Then, login and navigate to the UPLOAD tab, where you will find detailed instructions on how to use the tool. If you encounter any issues or have any questions when using PACE, please email us at figures@plos.org.

Data Requirements:

Please note that, as a condition of publication, PLOS' data policy requires that you make available all data used to draw the conclusions outlined in your manuscript. Data must be deposited in an appropriate repository, included within the body of the manuscript, or uploaded as supporting information. This includes all numerical values that were used to generate graphs, histograms etc.. For an example see here: http://www.plosbiology.org/article/info%3Adoi%2F10.1371%2Fjournal.pbio.1001908#s5.

Reproducibility:

To enhance the reproducibility of your results, we recommend that you deposit your laboratory protocols in protocols.io, where a protocol can be assigned its own identifier (DOI) such that it can be cited independently in the future. Additionally, PLOS ONE offers an option to publish peer-reviewed clinical study protocols. Read more information on sharing protocols at https://plos.org/protocols?utm_medium=editorial-email&utm_source=authorletters&utm_campaign=protocols

References

Please review your reference list to ensure that it is complete and correct. If you have cited papers that have been retracted, please include the rationale for doing so in the manuscript text, or remove these references and replace them with relevant current references. Any changes to the reference list should be mentioned in the rebuttal letter that accompanies your revised manuscript. If you need to cite a retracted article, indicate the article's retracted status in the References list and also include a citation and full reference for the retraction notice.

Attachment Submitted filename: PNTD-D-24-00259 FGS manuscript in Ghana (1) July 2024.pdf

# Author comment: Author response to Decision Letter 1

**DOI:** 10.1371/journal.pntd.0012443.r004

2 Aug 2024

Attachment Submitted filename: Cover letter_response to reviwers comments_01_08_2024.docx

# Editor report: Decision Letter 2

**DOI:** 10.1371/journal.pntd.0012443.r005

**Authors:** Guilherme L Werneck

7 Aug 2024

Dear Dr. Dalaba,

We are pleased to inform you that your manuscript 'Breaking the silence of female genital schistosomiasis in Ghana’s health system: A case of health workers within the FAST project' has been provisionally accepted for publication in PLOS Neglected Tropical Diseases.

Before your manuscript can be formally accepted you will need to complete some formatting changes, which you will receive in a follow up email. A member of our team will be in touch with a set of requests.

Please note that your manuscript will not be scheduled for publication until you have made the required changes, so a swift response is appreciated.

IMPORTANT: The editorial review process is now complete. PLOS will only permit corrections to spelling, formatting or significant scientific errors from this point onwards. Requests for major changes, or any which affect the scientific understanding of your work, will cause delays to the publication date of your manuscript.

Should you, your institution's press office or the journal office choose to press release your paper, you will automatically be opted out of early publication. We ask that you notify us now if you or your institution is planning to press release the article. All press must be co-ordinated with PLOS.

Thank you again for supporting Open Access publishing; we are looking forward to publishing your work in PLOS Neglected Tropical Diseases.

Best regards,

Guilherme L Werneck

Section Editor

PLOS Neglected Tropical Diseases

Elsio Wunder Jr

Section Editor

PLOS Neglected Tropical Diseases

***********************************************************

The authors have reviewed the manuscript and adequately addressed all the recommendations.

# Editor report: Acceptance letter

**DOI:** 10.1371/journal.pntd.0012443.r006

**Authors:** Guilherme L Werneck

17 Sep 2024

Dear Dr. Dalaba,

We are delighted to inform you that your manuscript, " Breaking the silence of female genital schistosomiasis in Ghana’s health system: A case of health workers within the FAST project ," has been formally accepted for publication in PLOS Neglected Tropical Diseases.

We have now passed your article onto the PLOS Production Department who will complete the rest of the publication process. All authors will receive a confirmation email upon publication.

The corresponding author will soon be receiving a typeset proof for review, to ensure errors have not been introduced during production. Please review the PDF proof of your manuscript carefully, as this is the last chance to correct any scientific or type-setting errors. Please note that major changes, or those which affect the scientific understanding of the work, will likely cause delays to the publication date of your manuscript. Note: Proofs for Front Matter articles (Editorial, Viewpoint, Symposium, Review, etc...) are generated on a different schedule and may not be made available as quickly.

Soon after your final files are uploaded, the early version of your manuscript will be published online unless you opted out of this process. The date of the early version will be your article's publication date. The final article will be published to the same URL, and all versions of the paper will be accessible to readers.

Thank you again for supporting open-access publishing; we are looking forward to publishing your work in PLOS Neglected Tropical Diseases. 

Best regards,

Shaden Kamhawi

co-Editor-in-Chief

PLOS Neglected Tropical Diseases

Paul Brindley

co-Editor-in-Chief

PLOS Neglected Tropical Diseases
